# Cheminformatics-driven discovery of natural isoquinoline alkaloid inhibitors of Beta-secretase I for Alzheimer’s management

**DOI:** 10.1371/journal.pone.0343717

**Published:** 2026-03-02

**Authors:** Iqra Ahmad, Sara Waheed, Bader S. Alotaibi, Sumra Wajid Abbasi, Muhammad Umer Khan, Faiz Ur Rahman, Hanna Dib, Khaled Fahmi Fawy, Umar Nishan, Abid Ali, Aqal Zaman, Mohibullah Shah

**Affiliations:** 1 Department of Biochemistry, Bahauddin Zakariya University, Multan, Pakistan; 2 Department of Clinical Laboratory Sciences, College of Applied Medical Sciences, Al- Quwayiyah, Shaqra University, Riyadh, Saudi Arabia; 3 Department of Biological Sciences, National University of Medical Sciences, Rawalpindi, Pakistan; 4 Institute of Molecular Biology and Biotechnology, The University of Lahore, Lahore, Pakistan; 5 Department of Zoology, University of Shangla, Swat, Khyber Pakhtunkhwa, Pakistan; 6 College of Engineering and Technology, American University of the Middle East, Kuwait; 7 Department of Chemistry, Faculty of Science, Research Center for Advanced Materials Science (RCAMS), King Khalid University, Abha, Saudi Arabia; 8 Department of Chemistry, Kohat University of Science & Technology, Kohat, Pakistan; 9 Department of Zoology, Abdul Wali Khan University Mardan, Mardan, Pakistan; 10 Department of Microbiology & Molecular Genetics, Bahauddin Zakariya University, Multan, Pakistan; 11 Department of Animal Science, Federal University of Ceara, Fortaleza, Brazil; Inonu University, Faculty of Pharmacy, TÜRKIYE

## Abstract

Alzheimer’s disease (AD) is characterized by the gradual deterioration of cognitive functions, speech impairment, and memory loss. It can potentially be treated by targeting the beta-site amyloid precursor protein cleavage enzyme 1 (BACE1), which plays a key role in amyloid plaque formation, neurofibrillary tangles, and hyperphosphorylated tau protein. Current drugs have limitations in terms of safety, efficacy, and blood-brain barrier permeability. In view of this, this study was designed to determine the potential inhibitors of the BACE1 enzyme by virtual screening using a curated library of 415 natural products including terpenoids, phenolic compounds, and alkaloids from different medicinal plants. Based on the docking score and interaction analysis, 50 compounds were selected for the downstream analysis, such as ligand binding interactions, pharmacokinetics, druglikness and physicochemical parameters. Among the lead compounds, Palmatine (compound 45) and Berberine (compound 49), demonstrated optimal drug-likeness and blood-brain barrier permeability among the top compounds. 2-[(9Z,12Z)-heptadeca-9,12-dienyl]-6-hydroxybenzoic acid (compound 4) was inactive in most toxicity parameters. Pharmacophore analysis revealed that Palmatine and Berberine share similar features with the standard, highlighting their potential as effective compounds. Furthermore, structural chemistry analysis provided insights on their shared isoquinoline alkaloid framework, illustrating their structural similarities. Molecular dynamics simulations confirmed the stability of the Palmatine-BACE1 and Berberine-BACE1 complexes during a 50 ns production run. Overall, these findings highlighted the potential of Palmatine and Berberine as promising candidates for the experimental validation and the development of the drugs for the treatment of AD.

## 1. Introduction

Alzheimer’s Disease (AD) is a chronic, neuronal loss-based age-related disease characterized by two major biomarkers, such as amyloid beta (Aβ_42_) and neurofibrillary tangles (NFTs), accompanied by synaptic degeneration and tau proteins. Several investigations have shown that AD is the most common cause of dementia and neuronal loss. The beta-secretase 1 (BACE1) enzyme cleaves amyloid precursor protein in its excessive form increasing beta-amyloid production [1]. These then accumulate in the form of amyloid plaques in the extracellular area between the neurons. These amyloid plaques disturb the neuronal signaling between neurons and cause impaired cognitive memory patterns. About a century after the discovery of this disease, only a few drugs have been approved by the U.S. Food and Drug Administration (FDA). These drugs, rivastigmine (Exelon), galantamine (Razadyne and Reminyl), tacrine (Cognex), and donepezil (Aricept), are based on the cholinergic hypothesis but devoid of amyloid hypothesis-based drug design. Cholinergic-based inhibitors reduce the symptoms of AD, whereas the etiology of the disease is not yet known [1]. Crucially, there are currently no FDA-approved BACE1 inhibitors for AD. This creates an urgent need to identify lead compounds that can effectively modulate BACE1. Since BACE1 knockout in mice reduces beta-amyloid formation [2], regulating its function remains crucial for resolving AD etiology.

The proper treatment of AD is still ambiguous, as in the recent decade, researchers have been widely investigating the associated risk factors, such as obesity, diabetes, vascular health, and circadian rhythm disruption [3]. Numerous synthetic BACE1 inhibitors have reached clinical trials; however, the majority failed due to off-target effects and dose-limiting toxicities [4]. An extensive analysis of the literature depicts that the physicochemical parameters, drug-likeness, and pharmacokinetics play an integral role in the treatment of AD. Among the pharmacokinetics, the blood-brain barrier (BBB) is the key factor to consider in neurological disorders. The blood-brain barrier is a semipermeable structural barrier that restricts certain substances from passing the blood to the brain. The integrity of the BBB is vital in AD pathogenesis; its compromise facilitates increased amyloid-beta accumulation and upregulated BACE1 activity, further accelerating neurodegeneration [5]. Research findings depict that 98% of small molecules cannot cross the BBB, and currently <1% of drug development is focused on its delivery to the Central Nervous System (CNS), has created an immense gap in AD etiology resolving therapeutics. AD treatment requires those non-toxic molecules with permeability to cross this barrier so that the desired drug can reach its target site in the brain [5].

Natural products hold immense potential as therapeutic agents against neurological disorders. However, identifying the ideal lead molecule from vast chemical spaces is a significant challenge. Cheminformatics provides a high-throughput, cost-effective platform to prioritize compounds with the highest probability of clinical success [6–9]. Three key classes of secondary metabolites, such as phenols, alkaloids and terpenoids, were concentrated on to curate the library of small molecules. Phenolic compounds such as flavonoids and lignans are present in various fruits, vegetables, and herbal infusions and have multifunctional agents that delay the progression of AD [10]. Alkaloids possess multiple actions such as anti-inflammatory, anti-cancer, and antibacterial, along with potential pharmacological actions in reducing neurodegenerative diseases [11–13]. Terpenoids act as promising agents in the treatment of neuropathy, wound healing, and its associated diseases [14].

This study aimed to design novel drugs through computational analysis by investigating the natural products from different plants and find potent lead molecules that could target BACE1 to reduce amyloid-beta production and also possess the best pharmacokinetics, specifically the ability to cross the blood-brain barrier, for the treatment of AD. The lead compounds suggested in this study have the potential to be considered as candidate molecules for validation through the experimental analysis for the drug designing against the AD.

## 2. Materials and methods

### 2.1. Retrieval of target protein

**Fig 1:**
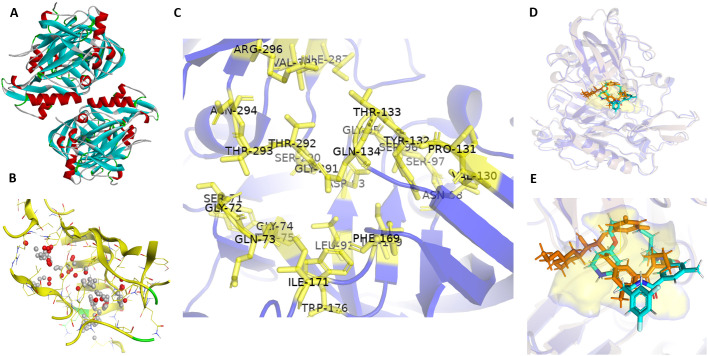
Structural characterization and active site analysis of Beta Secretase 1 (BACE1) for structure-based drug design. A) 3D structure of BACE1 (PDB ID 2QP8) with active site residues in chain A and B. B) Active site of BACE1; white spheres show hydrophobic residues whereas red spheres show hydrophilic residues, C) Zoomed-in view of the active site residues of BACE1. D) Superimposition of the BACE1 crystal structure (blue) and the docked protein (pink) showing the alignment of native and docked ligands. E) Zoomed-in view of the native ligand SCH734723 (orange) superimposed with its docked pose (cyan) within the active site surface (yellow).

The three-dimensional X-ray crystallographic structure of Beta Secretase 1 (BACE1) was retrieved from the RCSB protein data bank (https://www.rcsb.org/) with PDB ID 2QP8 (Fig 1A) [15]. This structure was selected due to its high resolution (1.50 Å), absence of mutations, and the presence of the co-crystallized inhibitor SCH734723 as a reference. This X-ray-resolved 3D structure of the protein was loaded and prepared as a receptor in the structure-based drug design approach. The co-crystallized ligand was removed during the structure preparation of proteins to create a vicinity at active sites for binding ligand molecules.

### 2.2. Identification of the active site

The active site of the target protein (BACE1) was determined using MOE based on alpha centers. The amino acids involved in the binding pocket interacting with ligands were determined using the Site Finder option in MOE [16]**.** Dummy atoms were created at those potential site points for the binding of ligands. White sphere atoms represent hydrophobicity and red color atoms show hydrophilicity of binding pocket residues shown in Fig 1B.

### 2.3. Ligands library preparation

We prepared a curated library of selected secondary metabolites isolated from different medicinal plants in the literature. These *in vitro* validated compounds were selected to check their potential interactions with BACE1. The database consists of 415 chemical compounds that belong to all three major classes of natural products, such as: 111 compounds are terpenoids, 126 are phenolic compounds, including flavonoids such as flavones, polyphenol lignans, flavanones, and chalcones, and the remaining were alkaloids, tannins, and glycoside derivatives (S1 Table). The 2D structure of the compounds present in our database was obtained from PubChem. PubChem is a database that generally provides two and three-dimensional structures of small and large molecules [17]. Drug-like chemical structures obtained from PubChem have been drawn in an offline software named CS ChemDraw Pro and saved in mol format to open in MOE. The energy minimization of the ligands was done using the default parameters of MOE. MK-8931 is an FDA-approved small-molecule BACE1 inhibitor that has decreased beta-amyloid cerebrospinal fluid levels by up to 84% in AD patients. This drug has entered the 2/3 phase of clinical trials and has shown efficacy, safety from toxicity, and well-tolerated results. Based on these facts, MK-8931 was selected as a control for the molecular docking study [18].

### 2.4. Validation of docking parameters

Before performing molecular docking, it’s crucial to validate the parameters and algorithms used in the docking process [19]. This ensures that the docking simulations are reliable and accurate. Self-docking was performed by re-docking the native ligand SCH734723, and interaction diagram analysis was compared with selected PDB literature. The reproducible similar interactions at the same binding position validated the docking parameters.

### 2.5. Molecular docking

The molecular operating environment software (Molecular Operating Environment [MOE], 2022.02 Chemical Computing Group ULC, 1010 Sherbrooke St. West, Suite #910, Montreal, QC, Canada, H3A 2R7, MOE2022.v11.18.1) was used to perform the docking. The structure of BACE1 was prepared by de-solvation to remove the water molecule, protonation to add hydrogen, and energy minimization to reduce steric hindrances and make the structures stable using default options in MOE. Before proceeding to dock, the energy of the ligands was also minimized to eradicate the number of steric clashes among the atoms of the ligand. The rigid water molecules were constrained by default. To determine the interaction between the receptor’s binding pocket and ligand, induced fit docking was done and 10 cycles were selected with other default options. Further, ligand-protein interactions were assessed using the PyMOL visualization system. The docking score of enzyme-ligand complexes and their three-dimensional structures were saved.

The estimated inhibition constant (Ki) for the lead compounds was calculated from the docking scores (ΔG) using the formula Ki = e^(ΔG/ RT)^, where R is the gas constant (1.987 × 10^−3^ kcal/mol·K) and T is 298.15 K [20].

### 2.6. Drug-like properties

Drug properties like the physicochemical properties of small molecules based on Lipinski’s, Ghose’s, and Veber’s rules were determined using the SwissADME online tool [21]. Lipinski’s rule of five for CNS active drugs was applied to determine the drug-like potential of small-molecule inhibitors. Lipinski’s rule evaluates the properties of a chemical compound, such as its biological activity, chemical properties, and physical properties, to determine whether it is an orally active drug. In order to assess a compound’s capacity to pass the blood-brain barrier and exhibit therapeutic effects inside the brain, adaptations of these criteria are taken into account for compounds intended to be active in CNS. These criteria include molecular weight <450 Daltons, Log P 2–5, H-bond acceptor <7, H-bond donor <3, and TPSA <60 Å^2^ [22]. According to Veber’s rule, the TPSA of the drug-like molecule must be ≤ 140 and a number of rotatable bonds<10. A Ghose’s filter was applied that determined the drug-likeness of molecules based on Molar refractivity (40–130), molecular weight (160–480), logP (−0.4–5.6), number of atoms (20–70), and TPSA< 140. Lipinski’s rule of five, Veber’s, and Ghose’s rule were applied to determine the potential candidate that could act as a drug-like ligand.

### 2.7. Pharmacokinetics analysis

In order to predict different pharmacokinetic properties of the ligands, including absorption, distribution, metabolism, excretion, and the blood-brain barrier of compounds in the human body, the SwissADME, an online tool was used [23].

### 2.8. Toxicity prediction

ProTox II is an online web server that describes toxicity based on LD50 [24]. Toxicity analysis of selective lead compounds was done to further confirm carcinogenicity using the online tool AdmetSAR [25].

### 2.9. Pharmacophore analysis

The pharmacophore matching approach was performed using Pharmacophore query editor of MOE [26] to find specific molecular properties of ligands involved in biological activity [27].

### 2.10. Molecular dynamics study

MD simulations were performed for the best lead compounds namely, palmatine and berberine models in complex with BACE1 due to their favorable pharmacokinetic properties compared to the standard using AMBER 18 [28] with previously defined parameters [29]. The complexes were chosen for coordinates at 50 ns for all atom molecular dynamic simulations. Using the Amber19 tools and the Antechamber program, the initial parameters and library files for proteins and ligands were created. ff14SB was employed for the protein, while the amber force field (GAFF) was chosen for the ligands [30]. The topology of the protein-ligand complexes was made using the Leap module of Amber19 tools. To balance the simulation systems, Na + was introduced. After being neutralized, the complexes were solvated with the aid of TIP3PBOX, a water molecule. Before starting the MD simulation production run, the solvated systems were carefully minimized. The steepest descent approach utilizing the SANDER module was used for the first 1500 iterations before switching to a conjugate gradient for the final 1000 steps. Steric clashes have been mitigated by doing these 2500 cycles of energy minimization. Each run initially included a 100-ps heating phase that steadily increased from 0 K to 300 K and 1 atm of pressure. A preliminary round of 100 ps of equilibration at a constant temperature of 300 K is required prior to the production phase. Kinetic and potential energies were swapped during the equilibration stage. The total energy remained relatively stable throughout the equilibration, although the potential and kinetic energies changed. A production run lasting 20 ns for each system was executed after equilibration to attain statistically reliable outcomes. All the atoms that were covalently bound to a hydrogen atom were constrained using the SHAKE method. Canonical ensembles and periodic boundary conditions were employed in the simulation box. In order to maintain a non-bonded cutoff of 8.0 and a constant temperature, the Berendsen coupling integration algorithm was applied. The Ewald summation approach was used to create MD simulations. Every 0.5 ps, the coordinate files were saved for the structural analysis. The CPPTRAJ module included in Amber18 was used to analyze the trajectories every 0.5 ps.

## 3. Results and discussion

### 3.1. Identification of the active site

MOE site finder Tool Predicted the possible active sites determined in the 3D atomic coordinates of the receptor BACE1. The active site of the protein was identified based on the binding site of the native ligand, and this identification was further supported by existing literature. The amino acid residues at the binding pocket of BACE1 were Ser71, Gly72, Gln73, Gly74, Tyr75, Leu91, Asp93, Gly95, Ser96, Ser97, Asn98, Val130, Pro131, Tyr132, Thr133, Gln134, Gly135, Phe169, Ile171, Trp176, Ile179, Ile287, Asp289, Ser290, Gly291, Thr292, Thr293, Asn294, Arg296, and Val393. These residues are shown in the binding pocket in Fig 1C.

### 3.2. Validation of the docking protocol

To verify the reliability and reproducibility of the docking algorithm, a self-docking (redocking) experiment was performed using the native inhibitor SCH734723. The native ligand was extracted and redocked into the active site of BACE1 (PDB ID: 2QP8). The docked pose demonstrated a high degree of structural homology with the original crystallographic orientation, yielding a Root Mean Square Deviation (RMSD) of 0.789 Å (Fig 1D and 1E). This low RMSD value, validates the docking protocol for the virtual screening of the natural product library.

### 3.3. Molecular docking analysis

The major underlying cause of AD is the deposition of amyloid beta plaques in neurons, which is caused by BACE1. By the clinical diagnosis of AD, patients typically exhibit a nearly twofold pathological upregulation of BACE1 protein levels and enzymatic activity in the brain. Therefore, it is an effective therapeutic strategy to target and control the expression of BACE1 [3]. Various therapeutic peptidomimetic inhibitors and antibodies have recently been designed but they failed in vivo due to their adverse side effects [31]. More than 60 percent of pharmaceuticals present in clinical and pre-clinical trials have been natural products since ancient times. The use of natural bioactive substances and their derivatives to treat AD is widespread because of their beneficial aspects and lack of adverse effects, but these small molecule BACE1 antagonists are also not long-lasting therapeutics due to poor blood-brain barrier penetration [5]. Therefore, we aimed to generate BBB permeant drug-like small molecules that could reach the central nervous system and reduce amyloid plaques by inhibiting BACE1. This study is focused on natural products such as terpenoids, flavonoids, and alkaloids to find potential drug candidates through the molecular docking approach. A curated library of secondary metabolites was prepared through a literature survey in which only *in vitro* validated inhibitors of BACE1 were collected, but their molecular interactions are not yet elucidated. These 415 compounds (S1 Table) were virtually screened against the target protein BACE1 to check the binding potential of these small molecules. Molecular docking was performed using the MMFF94x force field to screen small molecules, for which ten conformations were allowed per molecule and the highest docking score pose was selected. Based on the docking score generated during ligand-receptor binding with dummy atoms of the active site, 315 secondary products were found to be active, while 47 terpenoids, 12 flavonoids, 12 phenolic compounds, 09 alkaloids, 02 glycosides and 08 others were inactive and have shown no interactions with the Beta Secretase 1 receptor (S1 Table). The top 50 scoring compounds (S2 Table) were subjected to downstream analysis, such as ligand-binding interactions, pharmacokinetics, drug-likeness, and other physicochemical parameters. Of these, the two compounds, namely palmatine (compound 45) and berberine (compound 49), have shown the best drug-likeness-based physicochemical properties and BBB permeability, an integral property involved in treating neurological disorders [9].

The inhibitory potential was further quantified by calculating the estimated inhibition constants (Ki). The lead compounds exhibited Ki values in the range of 0.78 µM to 22.56 µM, as detailed in Table 1. Notably, melberrofuran G showed the highest potency (0.78 µM), while palmatine and berberine, demonstrated values of 19.50 µM and 22.56 µM, respectively. These values are consistent with potent natural product inhibitors and suggest a strong thermodynamic favorability for BACE1 binding.

**Table 1:** Molecular docking scores, estimated inhibition constants (Ki), and detailed binding interactions of the lead compounds within the BACE1 active site, observed in Molecular Operating Environment (MOE).

Compound Name	Docking Score (Kcal/mol)	Estimated Inhibition Constant (µM)	Residue	Interaction	Distance (Å)
Melberrofuran G	−8.3279	0.78	Val370: CA	Pi-H	4.49
Tellimagrandin I	−8.1479	1.06	Asp372: O	H-donor	3.24
			Phe220: O	H-donor	2.57
			Gly72: CA	H-acceptor	2.88
			Pro369: CD	Pi-H	4.32
Geraniin	−7.8132	1.87	Glu371: O	H-donor	2.76
			Gln134: OE1	H-donor	3.11
			Gly291: O	H-donor	2.98
			Gln73: OE1	H-donor	2.92
			Arg368: NH1	H-acceptor	3.38
			Thr293: N	H-acceptor	2.89
2-[(9Z,12Z)-heptadeca-9,12-dienyl]-6-hydroxybenzoic acid	−7.5967	2.69	Glu303: OE1	H-donor	2.84
1, 2, 3, 6-Tetra-O-galloyl-beta-D-glucose	−7.5729	2.81	Gly325: O	H-donor	3.14
			Pro369: O	H-donor	3.13
			Asp372: O	H-donor	3.07
			Arg368: NH1	H-acceptor	3.58
Sargachromenol	−7.5614	2.86	Asp372: O	H-donor	2.94
			Gly72: N	H-acceptor	2.75
Dieckol	−7.4846	3.26	Glu73: O	H-donor	2.60
			Gly173: O	H-donor	2.50
			Glu371: O	H-donor	2.70
Chrysophanol tetraglucoside	−7.4000	3.76	Pro396: O	H-donor	3.33
			Ser386: OG	H-acceptor	2.70
			Lys70: NZ	H-acceptor	2.71
			Arg368: NH1	H-acceptor	2.54
Neferine	−7.3946	3.79	Val373: O	H-donor	2.92
			Gln224: N	H-acceptor	3.00
			Lys70: NZ	Pi-cation	3.71
			Leu222: N	Pi-H	4.83
2-O-coumaryl-S-aloesinol	−7.2554	4.80	Pro131: OE1	H-donor	1.6
			Tyr259: O	H-donor	2.6
			Tyr259: O	H-donor	2.8
			Arg296: HH21	H-acceptor	2.4
Palmatine	−6.4249	19.50	Arg189: HH11	H-Acceptor	2.12
			Arg189: HH21	H-Acceptor	2.34
			Arg296: HH21	H-Acceptor	2.16
			Gly95: O	H-donor	2.51
			Thr390: O	H-donor	3.05
			Thr390: O	H-donor	2.97
			Thr390: OG1	H-donor	3.00
Berberine	−6.3386	22.56	Arg296: HH21	H-Acceptor	2.29
			Ser96: HB3	H-Acceptor	2.29
			Asn98: OD1	H-donor	2.31
			Asn98: OD1	H-donor	2.72
			Ile187: O	H-donor	2.40
			Thr390: O	H-donor	2.75
			Thr390: O	H-donor	2.97
			Thr390: OG1	H-donor	2.77

**Fig 2:**
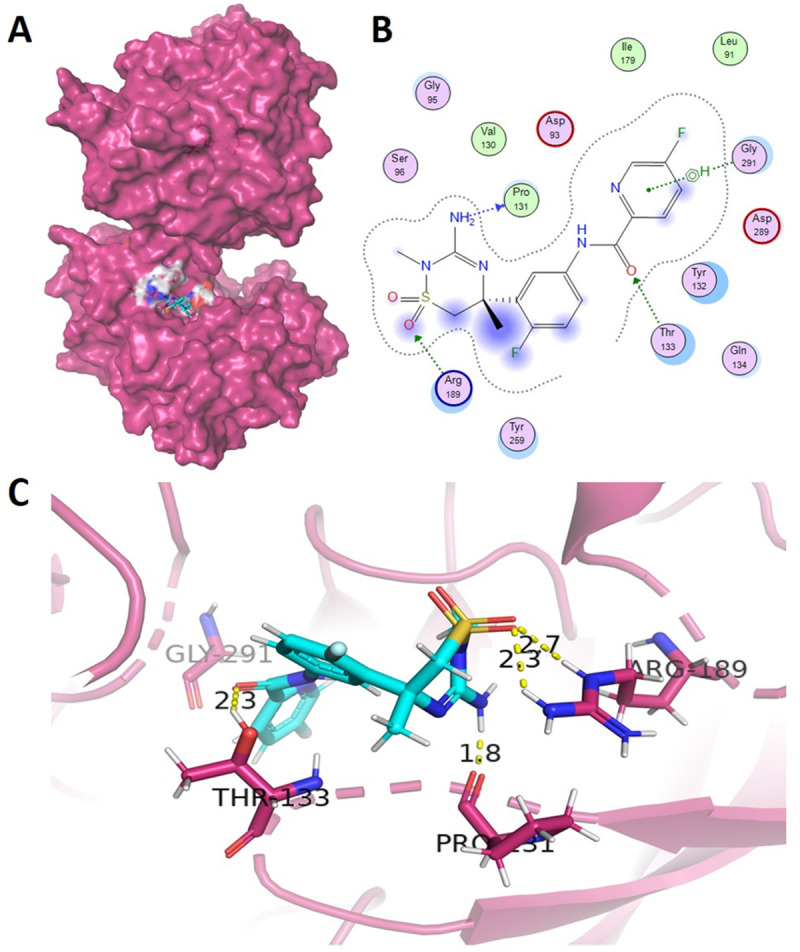
2D and 3D (A-C) interaction representation of standard (MK-8931) with BACE1.

Several hydrogen bonds were observed between the standard and the BACE1. Notably, the standard interacted with Pro131 through its N atom, establishing an H-donor interaction at a distance of 2.68 Å with an energy of −2.3 kcal/mol. One of the O atoms (from position 4) of the standard bonded with the NH2 atom of Arg189, acting as an H-acceptor at a distance of 2.98 Å with an energy of −2.0 kcal/mol. Another O atom of the standard (from position 6) formed a bond with Thr133 through its OG1, being an H-acceptor at a distance of 2.81 Å and having an energy of −0.9 kcal/mol. The standard also established a pi-H interaction with Gly291 using its 6-ring and CA atom from the receptor at a distance of 3.87 Å with an energy of −0.9 kcal/mol, observed in MOE, however, similar bonds were observed in PyMOL (Fig 2A-C).

**Fig 3:**
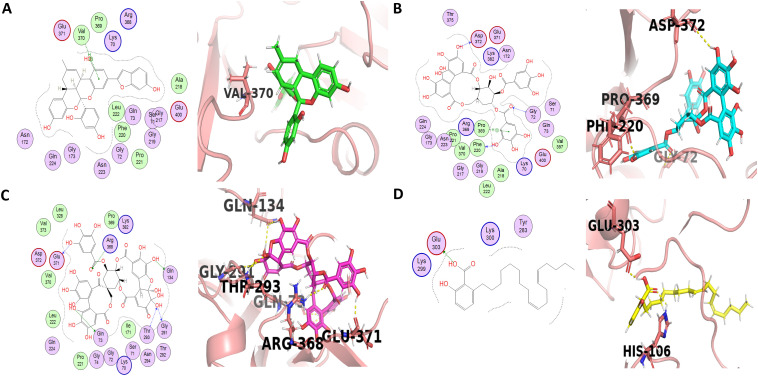
Ligand-protein interactions of four lead compounds (1-4) with with BACE1. Panel A-D shows 3D and 2D interactions of the corresponding ligands with the active site of BACE1. Melberrofuran G (compound 1), Tellimagrandin I (compound 2), Geraniin (compound 3) and 2-[(9Z,12Z)-heptadeca-9,12-dienyl]-6-hydroxybenzoic acid (compound 4).

The first active compound with the highest docking score of −8.3279 kcal/mol is Melberrofuran G (S2 Table), with its 6-ring form strong Pi-H bond with Val370 amino acid residue on CA receptor atom of A chain with released energy of −0.6 Kcal/mol and hydrophobic interaction with 19 different amino acid residues (Fig 3A). Moreover, it was previously reported that Melberrofuran G showed an inhibitory effect on BACE1 with an IC_50_ value of 0.3 μM [32].

Tellimagrandin I (Compound 2) was the second most active compound with a docking score of −8.1479 which was extracted from *Cornus officinalis* and formed three standard hydrogen bonds (two donors and one acceptor) with its terminal oxygen atom with Asp372 and Phe220 residues at the A chain of receptor atoms with releasing energies of −1.3Kcal/mol and −1.2Kcal/mol at interaction distances 3.24 and 2.57 respectively, and a hydrogen acceptor bond with Gly72 residue with receptor atom CA in chain A at distance 2.88 with releasing energy of −1.0 Kcal/mol. Tellimagrandin I showed another Pi-H bond forming between its 6-ring with the Pro369 residue and the CD receptor atom of chain A at an interaction distance of 4.32 with a binding energy of −0.6 Kcal/mol and hydrophobic interaction with 23 amino acid residues (Fig 3B). Moreover, in the literature, this compound had a borderline inhibitory effect on beta-secretase activity with an IC_50_ 52.82 ± 3.9 μM [33].

Geraniin (Compound 3) is the third active flavonoid compound extracted from *Geranium thunbergii* with docking score of −7.8132, forming four hydrogen donor bonds with Glu371, Gln134, Gly291, and Gln73 (Fig 3C) with energy released of −2.5 Kcal/mol, −1.1 Kcal/mol. −2.0 Kcal/mol and −0.8 Kcal/mol and interaction distances 2.76, 3.11, 2.98, and 2.92, respectively. A bond with Gly291 was also observed with the standard (Fig 2). The inhibitory activity of beta-secretase was moderate, with an IC_50_ of 4.0 × 10 − 6 M. It also formed two hydrogen acceptor bonds with Arg368 and Thr293 in chain A with binding energies of −2.2 Kcal/mol and −1.2 Kcal/mol at distances of 3.38 and 2.89, respectively. It also displayed hydrophobic interactions with 21 amino acid residues [34].

Succeeding Geraniin, the compound 4, 2-[(9Z,12Z)-heptadeca-9,12-dienyl]-6-hydroxybenzoic acid showed a docking score of −7.5967 by forming an H-donor interaction with Glu303 at chain A with a binding energy −5.2Kcal/mol and distance 2.84. It has also demonstrated hydrophobic interactions with 17 amino acid residues such as GlnB73, GlyB72, AsnB172, ileB171, LysB168, TyrA283, ProB105, HisB106, PheB108, ProB107, LysA300, GluA303, LysA299, SerB166, ThrB164, GluB165, PheB170 (Fig 3D, S2 Table). Moreover, in the literature, this compound has been extracted from *Homalomena occulta* with inhibitory activity on BACE1 7.9 μM 7.93 ± 0.38 [32].]

**Fig 4:**
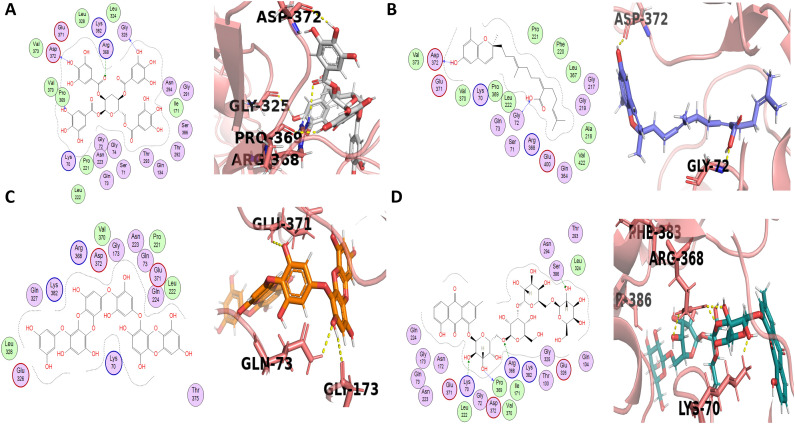
Ligand-protein interactions of four lead compounds (5-8) with BACE1. Panels A-D shows 3D and 2D interactions of the corresponding ligands with the active site of BACE1. 1, 2, 3, 6-Tetra-O-galloyl-beta-D-glucose (compound 5), Sargachromenol (compound 6), Dieckol (compound 7) and Chrysophanol tetraglucoside (compound 8).

1, 2, 3, 6-Tetra-O-galloyl-beta-D-glucose, the active compound 5 extracted from a marine sponge of the genus *Myrmekioderma*, demonstrated docking score −7.5729 by forming three H-donor bonds with Gly325, Pro369, and Asp372 at a distance of 3.14, 3.13, and 3.07 with released energy of −1.7, −1.3. −2.6, respectively, and an H-acceptor bond with Arg368 at a distance of 3.58 with the energy released −0.6 Kcal/mol. Moreover, this compound demonstrated hydrophobic interactions with 25 amino acid residues such as Gly325, Leu324, Leu328, Lys382, Glu371, Asp372, Val373, Arg368, Val370, Pro369, Asn294, Gly291, Thr292, Ile171, Ser386, Thr293, Gln73, Gly74, Asn223, Lys70, and Pro221 (Fig 4A, S2 Table). Among these hydrophobic interactions, Gly291 was also observed to be bounded (non-covalently) with the standard (Fig 2). Previous literature depicts that this compound exhibits weak inhibition activity on beta-secretase with an IC_50_ of 42.1 μM [33].]

Sargachromenol (Compound 6) from brown algae, *Sargassum serratifolium* inhibited beta-secretase with an IC_50_ value of 7.0 μM [32]. It has displayed a docking score of −7.5614 (S2 Table) and forms an H-donor and an H-acceptor bonds with Asp372 and Gly72, respectively. It also formed hydrophobic interactions with 20 amino acid residues (Fig 4B).

Dieckol (Compound 7) from *Ecklonia cava* exhibited a docking score of −7.4846 by forming three hydrogen bonds with Gln73, Gly173 and Glu371 (observed in PyMOL) with the bond distances of 2.6, 2.5 and 2.7 Å, respectively. It also interactions with 17 amino acid residues (Fig 4C). It had an IC_50_ value of 2.34 ± 0.10 µM in BACE1 inhibition [35,36] Chrysophanol tetraglucoside (Compound 8) from *Cassia obtusifolia* significantly inhibited beta-secretase activity, i.e., 26.19 ± 0.72 μg/Ml and displayed a docking score of −7.40 with three H-acceptor residues such as Ser386, Lys70, and Arg368. It also displayed an H-donor bond with Pro369 (Fig 4D). Thirdly, it has demonstrated hydrophobic interaction with 23 amino acid residues [37,38]

**Fig 5:**
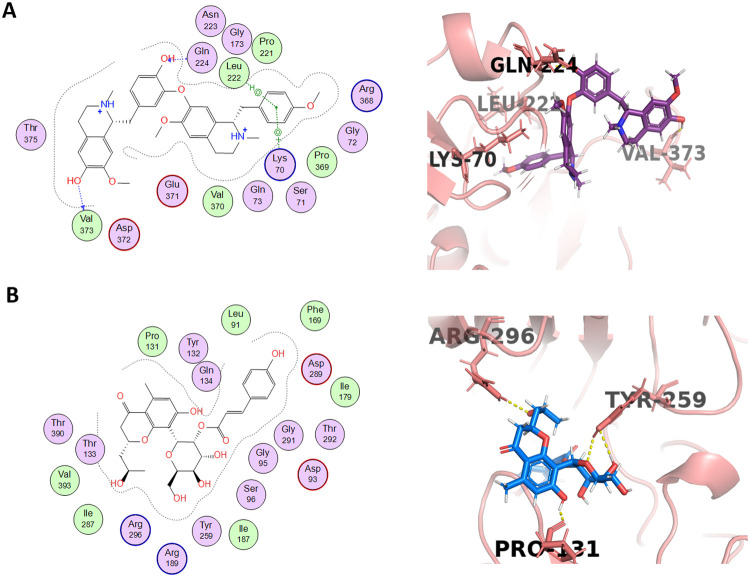
Ligand-protein interactions of two lead compounds (9-10) with BACE1. Panels A-B shows 3D and 2D interactions of the corresponding ligands with the active site of BACE1. Neferine (compound 9) and 2-O-coumaryl-S-aloesinol (compound 10).

Neferine (Compound 9) is an alkaloid isolated from *Nelumbo nucifera* that showed a docking score of −7.3946 by forming hydrophobic interactions with 16 amino acid residues (Fig 5A). It had shown inhibition activity on beta-secretase with IC_50_ values of 28.5 µM (Murata, 2019). 2-O-coumaryl-S-aloesinol (Compound 10) is a chromone glycoside extracted from Aloe vera and Aloe nobilis that presented a docking score of −7.2554 by forming four hydrogen bonds observed in PyMOL, with Pro131, Tyr259 and Arg296 (two bonds) with the bond distances of 1.6, 2.6. 2.8 and 2.4 Å, respectively as well as forming hydrophobic interactions with 19 amino acid (Fig 5B). Moreover, it has significant *in vitro* inhibitory activity on beta-secretase with an IC_50_ value of 20.5 µM [32].

**Fig 6:**
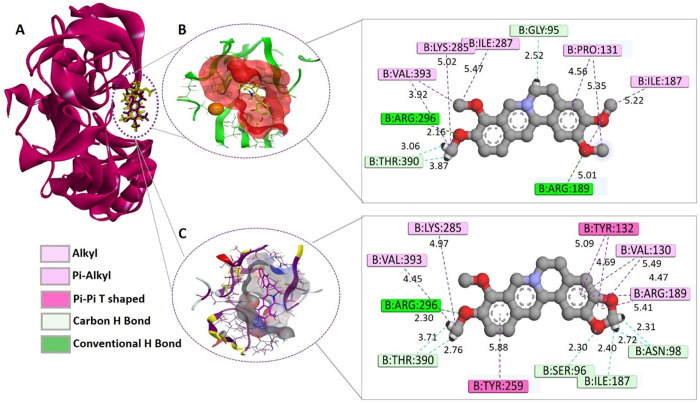
Binding interactions analysis of palmatine and berberine complexes with BACE1. A) Palmatine and berberine in complex with BACE1. B) 3D and 2D binding interaction of palmatine with BACE1. Palmatine (shown in yellow) molecular surface shown in red color. C) 3D and 2D binding interaction of berberine with BACE1. Berberine (shown in purple) hydrophobic interactions shown in grey.

Palmatine (Compound 45) bound strongly to the B chain of receptor BACE1 through five hydrogen bonds, with a docking score of −6.4249 kcal/mol. Two conventional hydrogen bonds were observed with Arg296 and Arg189 with the bond distances of 2.16 and 5.01 Å, respectively. Moreover, three carbon hydrogen bonds were observed with Gly95 and Thr390 (two bonds) with the bond distances of 2.52, 3.87 and 3.06 Å, respectively. However, alkyl interactions were also observed with several residues (Fig 6A and B). Palmatine showed hydrophobic interaction with BACE1–15 amino acid residues. In literature, palmatine possesses an inhibitory effect on beta-secretase activity with an IC_50_ value of 23.06 µM [39].

Berberine (Compound 49) displayed a strong hydrogen bonding by showing the tendency to form seven hydrogen bonds, with a docking score of −6.3386 Kcal/mol. It included one traditional hydrogen bond with Arg296 with the distance of 2.30 Å. Additionally, it included six carbon hydrogen bonds with Ser96, Asn98, Ile187 and Thr390 with variable bond distances (Fig 6C). Moreover, in the previous report, berberine also displayed an inhibitory effect on beta-secretase activity with an IC_50_ value of 28.70 mM [39].

**Fig 7:**
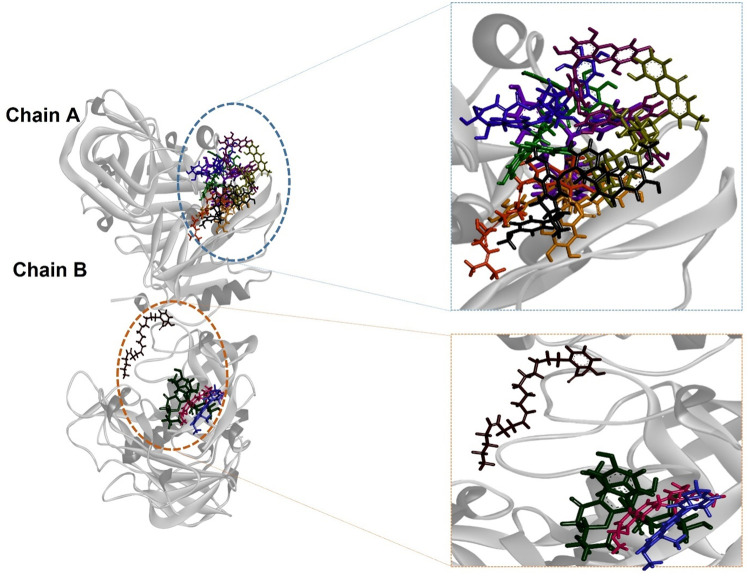
BACE1 with the lead compounds superimposed onto the respective binding sites. Eight and four ligands were bound to the active sites at chain A and B, respectively.

The binding positions of the top compounds on BACE1 were created by superimposing the ligands onto the protein structure (Fig 7). The placement of the ligands was based on their aforementioned interactions, ensuring an accurate representation of the binding sites. A cluster of ligands (compound 1–3 and 5–9) was observed to bind in close proximity to each other on chain A of the protein. Conversely, a separate cluster comprising compound 10, 45, and 49 was found to bind in close proximity on chain B. Notably, compound 4 exhibited binding to chain B; however, it occupied a distinct location compared to the other ligands on chain B (Fig 7B).

Hydrogen bonds, arene-H bonds, and hydrophobic interactions play a significant role in the stabilization of the protein-ligand complex. These interactions play an important role in predicting the best-fit molecules in the vicinity and catalytic pocket to control the dysregulated expression of BACE1. The docking score of all the described compounds and interactions with the receptor is better than the standard compound MK-8931, which reduces amyloid beta product with an IC_50_ of 13 nM [40].

Moreover, we examined the distribution of hydrogen bond numbers to provide a deeper understanding of the interaction dynamics. The distribution indicated that 50% of the compounds (compounds 2, 5, 7, 8, 9, and 10) form 4 hydrogen bonds, similar to the standard. Two compounds (compounds 4 and 6) formed two hydrogen bonds, and compounds 1, 45, and 49 formed distinct numbers of hydrogen bonds, specifically 1, 5, and 7 hydrogen bonds, respectively. This distribution of hydrogen bonds provides an average bond number of about 4 (3.92). Surprisingly, it can be seen that 4 hydrogen bonds are consistently repeating throughout the interaction profile with BACE 1. Moreover, compounds 3, 10, and 45 showed hydrogen bonding with residues similar to those in the standard.

Two lead compounds, palmatine and berberine, which exhibited higher docking scores than the standard compound, are isoquinoline alkaloids found in several plants and are known for their significant medicinal properties, including antimicrobial and anti-inflammatory activities [41]. They share a similar backbone structure, known as the isoquinoline skeleton, which contributes to their biological activities. Both molecules contain a quaternary ammonium group, a feature important for their interaction with BACE1 (Fig 6). However, a key difference lies in their substitution patterns on the isoquinoline ring system. Palmatine has a methoxy group that berberine lacks, which can influence their solubility, pharmacokinetics, and binding affinity to BACE1. This slight variation can lead to differences in their therapeutic effects and applications. Palmatine and berberine were highly emphasized in our study, due to their blood-brain barrier permeability predicted by the SwissADME profile.

Moreover, we also found that among the top compounds, neferine, palmatine and berberine have good GI absorption capabilities increasing their bioavailability. Pharmacophore analysis of the top 10 compounds is comparatively high, but palmatine and berberine also possess comparable features (hydrogen bond acceptor, hydrogen bond donor, hydrophobic and ionizable) to control the small molecule MK-8931. Similarly, palmatine and berberine showed perfect drug-like properties by following all drug-likeness rules (Ghose’s rule, Veber’s filter and Lipinski’s rule of five for CNS active drugs). Pharmacokinetics, pharmacophore analysis, and drug-likeness prediction revealed that the palmatine and berberine have similar physiochemical and molecular features compared to control inhibitor and could be used as potential drugs for AD. Results of this study exhibited that mostly phenolic compounds and a few alkaloids have higher inhibitory activity due to strong binding interactions than standard, and pharmacokinetics, pharmacophore analysis; drug-likeness prediction revealed that the palmatine and berberine have similar physiochemical and molecular features as compared with control inhibitors and could be used as potential drugs for AD. Since BACE1 possess a large open active site to strongly fit in the small molecule inside the pocket, which favors the binding of peptides as well, whereas studies reported the inefficacy of these drug-like properties on peptides *in vivo*, we deduce that small molecules following the drug like properties are the most potent compounds to further investigate in clinical trials.

### 3.4. Binding stability analysis through MD simulation

**Fig 8:**
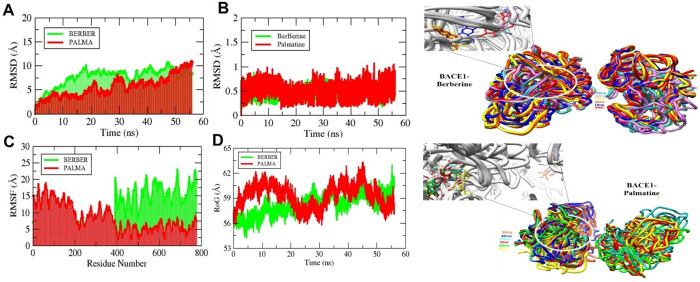
MD simulation trajectories are statistically analyzed. (A) Protein backbone RMSD (B) Ligand RMSD (C) RMSF and (D) ROG are the four output values. Binding conformation modifications of Berberine (Top) shown in different color sticks at the binding pocket of BACE1 protein (shown in grey color). Palmatine binding conformation changes (Bottom) shown as different coloured sticks in the BACE1 protein’s binding pocket (shown in grey color).

To check for structural stability and mobility throughout simulation, the fluctuations of the enzyme-ligand complex and the movements of the ligand inside the active site were examined in the hydrated environment. These movements are crucial to their operation and continued life inside the biological system, which guarantees their functional dependability and effectiveness. The ligand root mean square deviation (RMSD) value for palmatine and berberine remained around 1 Å. The protein backbone RMSD for both complexes fluctuated until ~ 10 Å (Fig 8A). Both compounds have shown relatively stable conformations with minor fluctuations whereas the overall observed RMSD was < 3.0 Å (Fig 8B) which means BACE1 has shown stable and stronger interaction with palmatine and berberine. Overall, the graphical presentation of compounds showed no major thermodynamics shifts and had stable performance (Fig 8A & B).

Root mean square fluctuation (RMSF) was analyzed to check the flexibility of α C atoms of active site residues in bound palmatine and berberine states. Berberine is highly stable at the active site (before residue number 400) residues as beta sheets and alpha helices didn’t undergo any major change whereas for palmatine, the RMSF value has shown significant fluctuations at active site residues because beta sheets tend to orient in order to fit in ligand interactions inside the active site pocket (Fig 8C).

The radius of gyration (RoG) over time in an MD simulation indicates conformational changes in macromolecules. RoG was observed to be more compact with highly ordered conformation remaining below 62 Å throughout the simulation (Fig 8D). This was a relatively smaller and more constant radius of gyration due to fewer conformational changes to adjust the ligand molecule inside the pocket.

### 3.5. Drug-like properties

Following the docking analysis, the top 50 compounds were screened to check for drug-likeness based on physicochemical properties. According to the Lipinski’s rule of five, small molecules that violate more than two rules cannot be used as an orally available drug. The molecular weight of a drug should not be higher than 450 g/mol, as it decreases the absorption rate. A lower consensus log P value (2–5) indicates higher hydrophilicity and thus higher absorption and permeation into the brain. The topologically polar surface area of the drug-like molecule should be lower than 60 Å^2^, showing better CNS penetration. Among the top ten compounds, 2-[(9Z,12Z)-heptadeca-9,12-dienyl]-6-hydroxybenzoic acid (compound 4) complied with the Lipinski’s rule of CNS active drugs with 2 violations (Table 2). Most of the small molecules among the top 50 compounds violate one or the other rule, whereas inhibitors such palmatine (Compound 45) and berberine (Compound 49) followed all the required physicochemical properties, including Lipinski’s, Veber’s, and Ghose’s filters which confer that they have the potential to be used as orally administered drugs (Table 2). This adherence not only highlights their potential as BACE1 inhibitors for Alzheimer’s disease therapy but also sets a benchmark for the oral bioavailability and safety profiles necessary in lead compounds. Their ability to meet these stringent criteria provides a significant advancement in Alzheimer’s drug discovery. Such results can lead to the development of treatments that are both effective and patient-friendly.

**Table 2:** Physicochemical parameters and drug-likeness of top compounds.

Compound No.	Physicochemical parameters								Drug likeness		
	MW	H-bond acceptors	H-bond donors	Rotatable bonds	MR	TPSA	Consensus Log P	Heavy atoms	Lipinski violations	Ghose #violations	Veber #violations
1	562.57	8	5	2	155.79	132.75	4.96	42	4	3	0
2	562.57	21	13	6	181.28	368.19	−0.05	56	4	3	1
3	562.57	25	14	3	210.48	431.79	−1.11	67	4	3	1
4	562.57	3	2	16	121.16	57.53	6.93	28	2	1	1
5	562.57	21	12	13	177.4	357.19	0.63	55	5	3	2
6	562.57	4	2	10	130.01	66.76	0.63	31	3	3	2
7	562.57	18	11	6	181.42	287.14	3.39	54	4	4	1
8	902.8	24	14	12	198.03	391.2	−4.81	63	5	4	2
9	610.74	8	2	9	183.55	83.86	5.19	45	2	3	0
10	544.55	11	6	8	137.58	183.21	1.25	39	4	3	1
11	785.01	13	9	10	204.64	218.99	2.4	55	5	3	1
12	686.61	14	7	13	176.13	229.74	3.26	50	5	3	2
13	566.51	10	4	5	155.91	159.8	4.39	42	4	3	1
14	680.7	9	7	6	192.61	160.07	5.55	51	3	4	1
15	940.68	26	15	16	214.27	444.18	−0.03	67	5	3	2
16	382.54	3	2	14	117.75	57.53	6.48	28	2	1	1
17	552.48	10	5	4	151.44	170.8	4.04	41	4	2	1
18	742.55	18	12	5	182.37	298.14	3.2	54	4	4	1
19	738.6	16	12	5	188.24	279.68	3.78	54	4	4	1
20	538.46	10	5	4	146.03	170.8	3.98	40	4	2	1
21	594.56	14	7	7	139.38	214.06	−0.45	42	4	4	1
22	936.69	25	15	9	216.99	434.95	0.05	67	4	3	1
23	442.37	10	7	4	110.04	177.14	1.36	32	3	0	1
24	596.53	15	8	7	140	238.2	−1.07	42	4	4	1
25	566.51	10	4	5	155.91	159.8	4.43	42	4	3	1
26	738.6	18	11	6	176.55	310.66	0.84	53	4	3	1
27	582.55	10	4	5	158.46	155.89	4.41	43	4	3	1
28	602.46	14	9	4	151.55	232.13	3.24	44	4	3	1
29	552.48	10	5	4	151.44	170.8	4.01	41	4	2	1
30	452.54	6	2	8	129.81	85.22	4.72	33	1	0	0
31	454.47	6	5	4	130.24	110.38	4.04	34	2	1	0
32	586.58	12	4	10	147.58	178.28	1.74	42	5	3	1
33	649.64	17	10	12	146.43	287.36	−3.74	45	5	4	2
34	558.53	8	5	2	156.08	132.75	5.26	42	3	3	0
35	506.59	9	2	6	129.58	144.27	2.43	36	2	2	1
36	566.51	10	4	5	155.91	159.8	4.34	42	4	3	1
37	735.73	19	10	15	169.13	305.82	−3.28	51	5	4	2
38	552.48	10	5	4	151.44	170.8	4.03	41	4	2	1
39	542.49	10	6	3	142.13	173.98	3.12	40	4	2	1
40	434.39	10	5	4	107.62	159.05	0.59	31	3	0	1
41	686.61	14	7	13	176.13	229.74	3.33	50	5	3	2
42	624.77	8	1	10	188.02	72.86	5.51	46	3	3	0
43	572.56	12	5	9	143.11	189.28	1.34	41	4	3	1
44	492.43	10	6	8	128.63	177.89	2.78	36	4	1	1
45	352.4	4	0	4	101.79	40.8	2.64	26	0	0	0
46	716.64	15	9	14	181.79	268.81	2.58	52	5	3	2
47	588.51	14	9	10	141.85	243.9	0.38	42	5	2	1
48	594.56	14	7	7	139.38	214.06	−0.65	42	4	4	1
49	336.36	4	0	2	94.87	40.8	2.53	25	0	0	0
50	536.49	9	5	3	149.91	161.57	4.30	40	3	3	1
**STANDARD**	409.41	7	2	4	105.82	126.13	1.43	28	1	0	0

### 3.6. Pharmacokinetics analysis

**Fig 9:**
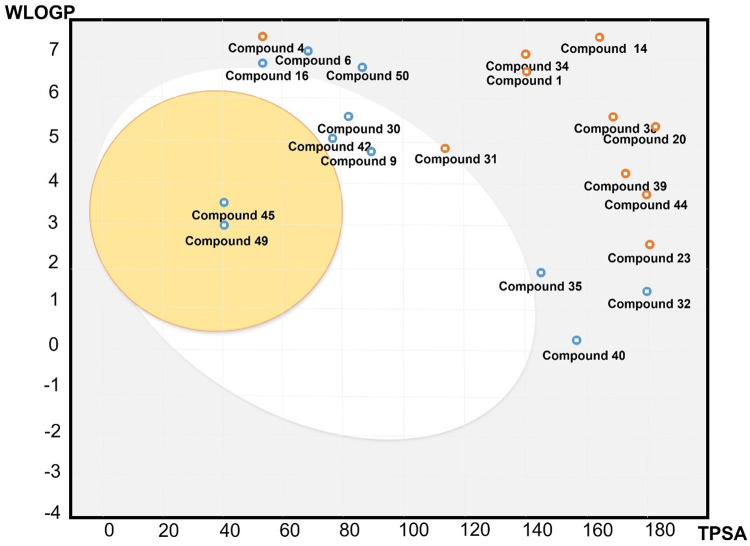
Pharmacokinetic analysis of top 50 compounds indicates palmatine (45) and berberine (49) within the BBB permeability range (Egg yolk), while others are outside this range (Egg white).

The pharmacokinetic analysis provides a pharmaceutical perspective on the viability of the lead compounds as orally active agents. The gastrointestinal (GI) absorption results of top compounds depict that Neferine, palmatine and berberine have high absorption in the GI tract (i.e., stomach, small intestine, and large intestine), which suggest their high oral bioavailability and ease of administration. The critical pharmaceutical challenge in AD is ensuring the drug reaches brain; SwissADME predicted that palmatine and berberine are two natural small molecules in the top 50 compounds that possess BBB permeability, correlating with their ability to follow the Lipinski’s rule of five criteria for CNS active drugs. This property demonstrates that potential palmatine and berberine drugs can be delivered across the BBB and treat neurodegenerative disorders. The boiled egg diagram also suggests that the palmatine and berberine have optimal lipophilicity and polarity to cross the BBB (Fig 9).

P-glycoprotein is highly expressed in the BBB and acts as an efflux transporter in the brain, where drug-like molecules that are active Pgp substrates are transported out of the brain, leading to therapeutic failure [42–44]. Melberrofuran G (compound 1), 2-[(9Z,12Z)-heptadeca-9,12-dienyl]-6-hydroxybenzoic acid (compound 4), Dieckol (compound 7) and Neferine (compound 9) were found to be inactive with Pgp (Table 3). The moderate solubility (ESOL Class) of the compounds: Tellimagrandin I (compound 2), Geraniin (compound 3), 1, 2, 3, 6-Tetra-O-galloyl-beta-D-glucose (compound 5), Sargachromenol (compound 6), palmatine (compound 45) and berberine (compound 49) ensures a steady-state concentration required for therapeutic efficacy at the BACE1 target site. Palmatine and Barbarine both were observed to be CYP2D6 and CYP3A4 inhibitors. While Chrysophanol tetraglucoside (compound 8) and 2-O-coumaryl-S-aloesinol (compound 10) were comparatively more soluble, which ensures the higher absorption (Table 3). These parameters collectively suggest that Palmatine and Berberine are not just potent inhibitors, but also pharmacologically viable candidates for CNS delivery.

**Table 3:** Pharmacokinetics parameters of top 50 compounds.

Compound No.	GI absorption	BBB permeant	Pgp substrate	log Kp (cm/s)	ESOL Class	CYP1A2 inhibitor	CYP2C19 inhibitor	CYP2C9 inhibitor	CYP2D6 inhibitor	CYP3A4 inhibitor
1	Low	No	No	−5.49	Poorly soluble	No	No	No	No	No
2	Low	No	Yes	−9.79	Moderately soluble	No	No	No	No	No
3	Low	No	Yes	−12.17	Moderately soluble	No	No	No	No	No
4	Low	No	No	−2.1	Poorly soluble	Yes	No	Yes	No	No
5	Low	No	Yes	−9.24	Moderately soluble	No	No	No	No	No
6	Low	No	Yes	−9.24	Moderately soluble	No	No	No	No	No
7	Low	No	No	−7.37	Poorly soluble	No	No	Yes	No	No
8	Low	No	Yes	−15.09	Very soluble	No	No	No	No	No
9	High	No	No	−5.5	Poorly soluble	No	No	No	No	No
10	Low	No	Yes	−8.63	Soluble	No	No	No	No	No
11	Low	No	Yes	−8.24	Poorly soluble	No	No	No	No	No
12	Low	No	No	−7.14	Poorly soluble	No	No	Yes	No	No
13	Low	No	No	−5.72	Poorly soluble	No	No	Yes	No	No
14	Low	No	No	−5	Poorly soluble	No	No	No	No	No
15	Low	No	Yes	−9.49	Poorly soluble	No	No	No	No	No
16	High	No	Yes	−2.04	Poorly soluble	Yes	Yes	No	Yes	No
17	Low	No	No	−5.86	Poorly soluble	No	No	Yes	No	No
18	Low	No	No	−7.55	Poorly soluble	No	No	Yes	No	No
19	Low	No	No	−6.97	Poorly soluble	No	No	No	No	No
20	Low	No	No	−5.57	Poorly soluble	No	No	Yes	No	No
21	Low	No	Yes	−10	Soluble	No	No	No	No	No
22	Low	No	Yes	−9.88	Poorly soluble	No	No	No	No	No
23	Low	No	No	−7.91	Soluble	No	No	No	No	No
24	Low	No	Yes	−10.63	Soluble	No	No	No	No	No
25	Low	No	No	−5.72	Poorly soluble	No	No	No	No	No
26	Low	No	No	−9.17	Moderately soluble	No	No	No	No	No
27	Low	No	No	−5.75	Poorly soluble	No	No	Yes	No	No
28	Low	No	No	−6.67	Poorly soluble	No	No	Yes	No	No
29	Low	No	No	−5.86	Poorly soluble	No	No	Yes	No	No
30	High	No	Yes	−4.89	Poorly soluble	No	No	Yes	No	Yes
31	High	No	No	−5.24	Poorly soluble	No	No	Yes	No	No
32	Low	No	Yes	−8.99	Soluble	No	No	No	No	No
33	Low	No	Yes	−14.22	Highly soluble	No	No	No	No	No
34	Low	No	No	−4.93	Poorly soluble	No	No	No	No	No
35	Low	No	Yes	−8.33	Soluble	No	No	No	No	Yes
36	Low	No	No	−5.72	Poorly soluble	No	No	Yes	No	No
37	Low	No	Yes	−14.48	Very soluble	No	No	No	No	No
38	Low	No	No	−5.86	Poorly soluble	No	No	Yes	No	No
39	Low	No	No	−6.42	Poorly soluble	No	No	Yes	No	No
40	Low	No	Yes	−8.11	Soluble	No	No	No	No	No
41	Low	No	No	−7.14	Poorly soluble	No	No	Yes	No	No
42	High	No	No	−5.35	Poorly soluble	No	No	No	No	No
43	Low	No	Yes	−9.14	Soluble	No	No	No	No	No
44	Low	No	No	−6.41	Moderately soluble	No	No	Yes	No	No
**45**	**High**	**Yes**	**Yes**	**−5.79**	**Moderately soluble**	**No**	**No**	**No**	**Yes**	**Yes**
46	Low	No	No	−8.08	Poorly soluble	No	No	No	No	No
47	Low	No	Yes	−8.87	Moderately soluble	No	No	No	No	No
48	Low	No	Yes	−10.4	Soluble	No	No	No	No	No
**49**	**High**	**Yes**	**Yes**	**−5.78**	**Moderately soluble**	**Yes**	**No**	**No**	**Yes**	**Yes**
50	High	Yes	Yes	−5.78	Moderately soluble	Yes	No	No	Yes	Yes

### 3.7. Toxicity results

The ProTox II web server predicted toxic properties of a small molecule such as hepatotoxicity, carcinogenic toxicity, immunotoxicity, mutagenicity, and cytotoxicity to save the drug from failure at clinical trials during the drug development process. While, none of the top compounds showed hepatotoxicity and cytotoxicity, most of the top compounds showed Immunotoxicity. Only Melberrofuran G (compound 1) showed carcinogenicity and only Chrysophanol tetraglucoside (compound 8) showed mutagenicity (Table 4). LD50 is a measure of toxicity, and the lower the LD50, the more toxic the small molecule is. Ideally, the value should be between 2000 and 5000 Kg/mol, which was followed by compound 2, 5 and 8 among the top ten compounds. Palmatine and berberine do not possess hepatotoxicity, whereas there is a minor probability of toxicity as compared to the standard molecule, as shown in (Table 4). To double check the carcinogenicity, AdmetSAR analysis was done, and it predicted the non-carcinogenicity and P-glycoprotein (Pgp) non substrate features of palmatine and berberine (S3 Table). To our knowledge, this is the first study that predicts two small molecules, i.e., palmatine and berberine that not only cross the BBB but are also inactive substrates of P-glycoprotein, endorsing brain delivery and overcoming drug resistance.

**Table 4:** Toxicity prediction and probability of top 50 compounds.

Compound No.	Hepatotoxicity	Carcinogenicity	Immunotoxicity	Mutagenicity	Cytotoxicity	LD50 Value	Toxicity class
1	Inactive 0.68	Active 0.57	Active 0.98	Inactive 0.62	Inactive 0.82	500	4
2	Inactive 0.81	Inactive 0.71	Active 0.61	Inactive 0.54	Inactive 0.81	3700	5
3	Inactive 0.75	Inactive 0.50	Active 0.99	Inactive 0.63	Inactive 0.71	620	4
4	Inactive 0.76	Inactive 0.74	Inactive 0.54	Inactive 0.82	Inactive 0.76	1000	4
5	Inactive 0.81	Inactive 0.65	Inactive 0.85	Inactive 0.87	Inactive 0.83	2260	5
6	Inactive 0.76	Inactive 0.64	Active 0.73	Inactive 0.83	Inactive 0.84	750	4
7	Inactive 0.78	Inactive 0.64	Inactive 0.94	Inactive 0.55	Inactive 0.91	866	4
8	Inactive 0.87	Inactive 0.88	Active 0.99	Active 0.63	Inactive 0.70	3000	5
9	Inactive 0.96	Inactive 0.59	Active 0.99	Inactive 0.52	Inactive 0.68	1180	4
10	Inactive 0.80	Inactive 0.84	Active 0.99	Inactive 0.59	Inactive 0.62	25	2
11	Inactive 0.94	Inactive 0.74	Active 0.99	Inactive 0.91	Inactive 0.88	4000	5
12	Inactive 0.65	Inactive 0.61	Active 0.92	Active 0.52	Inactive 0.77	25	2
13	Inactive 0.79	Inactive 0.67	Active 0.93	Inactive 0.80	Inactive 0.93	4000	5
14	Inactive 0.65	Active 0.53	Active 0.75	Inactive 0.58	Inactive 0.88	500	4
15	Inactive 0.80	Inactive 0.68	Inactive 0.91	Inactive 0.89	Inactive 0.82	2260	5
16	Inactive 0.80	Inactive 0.65	Active 0.86	Inactive 0.81	Inactive 0.93	4000	5
17	Inactive 0.73	Inactive 0.67	Inactive 0.88	Inactive 0.70	Inactive 0.93	3600	5
18	Inactive 0.79	Inactive 0.70	Active 0.91	Inactive 0.68	Inactive 0.81	1500	4
19	Inactive 0.75	Inactive 0.71	Inactive 0.93	Inactive 0.66	Inactive 0.91	4000	5
20	Inactive 0.83	Inactive 0.93	Inactive 0.99	Active 0.77	Active 0.50	2300	5
21	Inactive 0.73	Inactive 0.67	Inactive 0.88	Inactive 0.70	Inactive 0.93	3600	5
22	Inactive 0.79	Inactive 0.70	Active 0.91	Inactive 0.68	Inactive 0.81	1500	4
23	Inactive 0.75	Inactive 0.71	Inactive 0.93	Inactive 0.66	Inactive 0.91	4000	5
24	Inactive 0.83	Inactive 0.93	Active 0.99	Inactive 0.77	Active 0.50	2300	5
25	Inactive 0.81	Inactive 0.66	Inactive 0.67	Inactive 0.58	Inactive 0.77	3700	5
26	Inactive 0.83	Inactive 0.75	Active 0.98	Inactive 0.68	Inactive 0.76	2190	5
27	Inactive 0.75	Inactive 0.68	Active 0.98	Inactive 0.82	Inactive 0.84	5000	5
28	Inactive 0.75	Inactive 0.50	Inactive 0.95	Inactive 0.53	Inactive 0.91	280	3
29	Inactive 0.83	Inactive 0.75	Active 0.98	Inactive 0.68	Inactive 0.76	2190	5
30	Inactive 0.62	Inactive 0.67	Active 0.99	Inactive 0.70	Inactive 0.78	2000	4
31	Inactive 0.61	Active 0.52	Active 0.93	Inactive 0.58	Inactive 0.86	500	4
32	Inactive 0.78	Inactive 0.86	Active 0.99	Inactive 0.65	Inactive 0.63	25	2
33	Inactive 0.77	Inactive 0.77	Active 0.95	Inactive 0.73	Inactive 0.70	2000	4
34	Inactive 0.76	Active 0.55	Active 0.83	Inactive 0.70	Inactive 0.88	5000	5
35	Inactive 0.77	Inactive 0.55	Inactive 0.92	Inactive 0.86	Inactive 0.79	1500	4
36	Inactive 0.79	Inactive 0.67	Active 0.90	Inactive 0.80	Inactive 0.93	4000	5
37	Inactive 0.73	Inactive 0.69	Active 0.99	Inactive 0.73	Inactive 0.66	680	4
38	Inactive 0.80	Inactive 0.65	Active 0.92	Inactive 0.81	Inactive 0.93	3919	5
39	Inactive 0.73	Inactive 0.68	Inactive 0.73	Inactive 0.73	Inactive 0.81	2000	4
40	Inactive 0.87	Inactive 0.91	Active 0.51	Active 0.65	Inactive 0.59	5000	5
41	Inactive 0.65	Inactive 0.58	Active 0.97	Active 0.53	Inactive 0.77	25	2
42	Inactive 0.95	Inactive 0.59	Active 0.99	Active 0.54	Inactive 0.58	1180	4
43	Inactive 0.78	Inactive 0.86	Active 0.99	Inactive 0.65	Inactive 0.63	25	2
44	Inactive 0.62	Inactive 0.58	Active 0.98	Active 0.53	Inactive 0.80	2160	5
**45**	**Inactive 0.77**	**Active 0.50**	**Active 0.96**	**Active 0.57**	**Active 0.56**	**200**	**3**
46	Inactive 0.64	Inactive 0.60	Active 0.89	Active 0.55	Inactive 0.79	25	2
47	Inactive 0.81	Inactive 0.75	Inactive 0.64	Inactive 0.84	Inactive 0.83	2300	5
48	Inactive 0.81	Inactive 0.93	Active 0.99	Inactive 0.90	Inactive 0.52	2300	5
**49**	**Inactive 0.82**	**Active 0.56**	**Active 0.99**	**Active 0.62**	**Active 0.96**	**200**	**3**
50	Active 0.69	Inactive 0.62	Active 0.96	Inactive 0.97	Inactive 0.93	1190	4
Standard MK-8931	Inactive 0.52	Inactive 0.55	Inactive 0.88	Inactive 0.68	Inactive 0.64	650	4

### 3.8. Pharmacophore analysis

**Fig 10:**
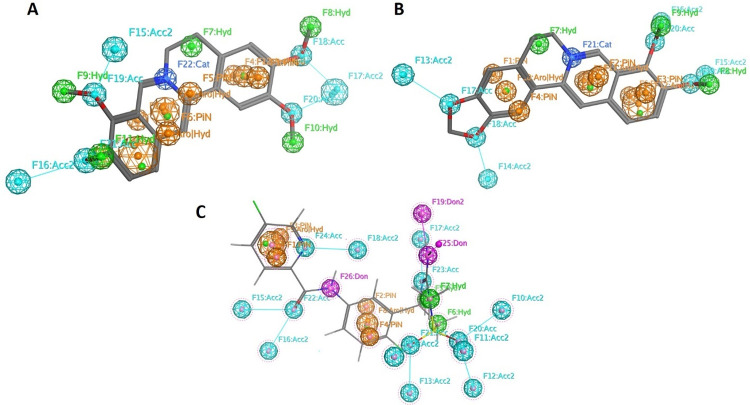
Pharmacophoric features of palmatine (A), berberine (B) and MK-8931 (C). Acc (hydrogen acceptor), Don (hydrogen donor), Hyd (hydrophobic), Aro (aromatic), PiN (Pi Network), and Cat (cationic interaction).

In pharmacophore modeling, various features were manually selected to represent the spatial arrangement of chemical groups important for the biological activity of palmatine and berberine. These features included PiN (Pi Network), Aro (aromatic), Hyd (hydrophobic), Acc (hydrogen bond acceptors), Cat (cationic) as annotated in Fig 10.

There were six PiN areas (F1 to F6), five potential sites for hydrophobic interactions (F7 to F10), three sites for combined possibility for aromatic and hydrophobic interactions (F12 to F14), three sites to form two hydrogen acceptor bonds (F15 to F17), four sites to form one hydrogen bond (F18 to F21), and finally, one cationic site (F22) were identified in Palmatine (Fig 10A). The selection of these features of berberine and palmatine reveals the common protoberberine nucleus shared by both alkaloids, with subtle differences observed in their interaction profiles (Fig 10B). Specifically, berberine exhibits two fewer hydrophobic interactions and possesses an additional site capable of forming dual hydrogen bond acceptor interactions compared to palmatine.

Pharmacophore analysis of these two compounds by MOE pharmacophore query deciphered that their pharmacophore features are comparable to the standard small molecule MK-8931 [18], except for the three potential features for hydrogen donor interactions in MK-8931 (Fig 10C). This similarity in the pharmacophoric features further evidentiate that the lead compounds described in this study have the full potential to be subjected to *in vitro* and *in vivo* studies for discovery of anti-AD.

While currently approved FDA treatments are primarily symptomatic and often limited by systemic side effects, the natural leads identified in this study, Palmatine and Berberine, offer a targeted disease-modifying alternative by inhibiting BACE1. These alkaloids provide a strategic advantage over existing synthetic drugs due to their predicted safety profiles and natural origin, offering a potential alternative to toxic synthetic drugs. Under the scope of this study, these identified lead compounds demonstrate the necessary BBB permeability without high P-gp efflux. However, this study may not capture the full biological complexity of Alzheimer’s disease, and the findings are preliminary utilizing only computer aided drug design approach. As a next step, it is imperative to experimentally validate the pharmacological effects of lead compounds, i.e., palmatine and berberine. Although these compounds adhered to the critical requirements for effective oral absorption and blood-brain barrier penetration, some strategies for lead optimization might needed. Moreover, the future work should also aim to study their metabolism and clearance rates, and their impact on cognitive functions in applicable disease models. Future studies should also focus on broadening the compound library to encompass a wider variety of chemical entities, potentially revealing new scaffolds for BACE1 inhibition.

## 4. Conclusion

Two distinct aspects were observed in this study, i.e., competitive inhibition by providing insights about the binding interaction of database ligands with the BACE1 binding site and the delivery of potential small molecules to the central nervous system. We investigated three important classes of selected natural products, and our findings suggested Melberrofuran G, Tellimagradin I, Geraniin, 2-[(9Z,12Z)-heptadeca-9,12-dienyl]-6-hydroxybenzoic acid, 1,2,3,6-Tetra-O-galloyl-beta-D-glucose, Sargachromenol, Dieckol, Chrysophanol tetraglucoside, Neferine, and 2-O-coumaryl-S-aloesinol have the potential to inhibit the activity of BACE1. Among the leading compounds, showing docking score higher than the standard, 2-[(9Z,12Z)-heptadeca-9,12-dienyl]-6-hydroxybenzoic acid (compound 4), was inactive in almost all toxicity parameters, and Palmatine (compound 45) and Berberine (compound 49) complied with Lipinski’s rule of Central Nervous System active drugs. MD simulation results further validated the stable binding of Palmatine and Berberine with BACE1 indicating that they have therapeutic potential to control BACE1 activity. These top compounds are highly selective in our study due to their best pharmacokinetics and physicochemical characteristics. Significant binding affinity, stable binding modes with BACE1, drug-likeness, inactive p-glycoprotein substrate, and most of all, BBB permeability are the attributes that provide valuable benchmarks for Palmatine and Berberine to further investigate for the treatment of AD.

## Supporting information

S1 Table Table shows the curated library of the selected natural compounds, their respective class, docking score and interacting residues of BACE 1. (DOCX)

S2 Table Table shows docking results, interacting residues of the receptor protein, nature, structure and names of the top 50 scoring compounds. (DOCX)

S3 Table Toxicity analysis of selective compounds by admetSAR. (DOCX)
