# Supplementary material for: Cheminformatics-driven discovery of natural isoquinoline alkaloid inhibitors of Beta-secretase I for Alzheimer’s management
Source: PLoS One. 2026 Mar 2;21(3):e0343717. doi: 10.1371/journal.pone.0343717 (PMC12952630; doi:10.1371/journal.pone.0343717)
Supplement: S1 Table — (DOCX) [file pone.0343717.s001.docx]

**Table S1.** Table shows the in-house library of the selected natural compounds, their respective class, docking score and interacting residues of BACE 1.

| **Sr. No.** | **Compound structure and Name** | **Docking score**  **(kcal/mol)** | **Chemical Nature/Class** | **Reference** |
| --- | --- | --- | --- | --- |
|  |   pterosinone | ­-3.8379 | Terpenoids | (1) |
|  |   Pteroside N | INACTIVE | Terpenoids | (1) |
|  |   Alpha turmerone | -4.4796 | Terpenoids | (2) |
|  |   Beta turmerone | -3.9472 | Terpenoids | (2) |
|  |   Ar turmerone | -4.7211 | Terpenoids | (2) |
|  |   L-zingiberene | -4.4088 | Terpenoids | (2) |
|  |   beta-sesquiphellandrene | -4.9997 | Terpenoids | (2) |
|  |   Ar- curcumene | -4.3921 | Terpenoids | (2) |
|  |   Loganin | -5.6658 | Terpenoids | (3) |
|  | Alpha –caryophyllene | INACTIVE | Terpenoids | (4) |
|  |   beta-caryophyllene | INACTIVE | Terpenoids | (4) |
|  |   beta-caryophyllene oxide | INACTIVE | Terpenoids | (4) |
|  |   Piperine | -4.5824 | Terpenoids | (4) |
|  |   Withanolide A | -4.6792 | Terpenoids | (5) |
|  |   Pistagremic acid | -4.5162 | Terpenoids | (6) |
|  |   Geranyl acetone | -4.0643 | Terpenoids | (7) |
|  |   positive camphor | INACTIVE | Terpenoids | (7) |
|  |   negative camphor | INACTIVE | Terpenoids | (7) |
|  |   Negative fenchone | INACTIVE | Terpenoids | (7) |
|  |   Positive fenchone | INACTIVE | Terpenoids | (7) |
|  |   ***ent*-Pimara-8(14),15-diene-19-oic acid** | -4.3423 | Terpenoids | (8) |
|  |   **18-nor-*ent*-Pimara-8(14),15-diene-4**β**-ol** | -3.3814 | Terpenoids | (8) |
|  |   ***ent*-Pimara-8(14),15-diene-19-ol** | INACTIVE | Terpenoids | (8) |
|  |   **7**α**-Hydroxy-*ent*-pimara-8(14),15-diene-19-oic acid** | -4.2474 | Terpenoids | (8) |
|  |   7β**-Hydroxy-*ent*-pimara-8(14),15-diene-19-oic acid** | -4.2193 | Terpenoids | (8) |
|  |   **7-Oxo-*ent*-pimara-8(14),15-diene-19-oic acid** | -4.4185 | Terpenoids | (8) |
|  |   ***ent*-Pimar-15-en-8**α**,19-diol** | INACTIVE | Terpenoids | (8) |
|  |   ***ent*-Kaur-16-en-19-oic-acid** | -3.4767 | Terpenoids | (8) |
|  |   **18-nor-*ent*-Kaur-16-ene-4**β**-ol (4-epiruilopeziol** | -3.3814 | Terpenoids | (8) |
|  |   **16**α**-Hydroxy-17-isovaleroyloxy-*ent*-kauran-19-oic**  **Acid** | -5.2467 | Terpenoids | (8) |
|  |   **16**α**,17-Dihydroxy-*ent*-kauran-19-oic acid** | -4.3571 | Terpenoids | (8) |
|  |   **16α-methoxy-17-hydroxy-*ent*-kauran-19-oic**  **Acid** | -4.7752 | Terpenoids | (8) |
|  |   **17-Hydroxy-*ent*-kaur-15-en-19-oic acid** | -4.4813 | Terpenoids | (8) |
|  |   **15**α**,16**α**-Epoxy-17-hydroxy-*ent*-kauran-19-oic** | INACTIVE | Terpenoids | (8) |
|  |   α-terpinyl acetate | -3.0378 | Terpenoids | (9) |
|  |   linaly acetate | -3.6946 | Terpenoids | (9) |
|  |   cinnamaldehyde | INACTIVE | Terpenoids | (9) |
|  |   pipataline | -3.9483 | Terpenoids | (9) |
|  |   safrol | INACTIVE | Terpenoids | (9) |
|  |   eugenol | INACTIVE | Terpenoids | (9) |
|  |   epicurzerenone | -3.7346 | Terpenoids | (9) |
|  |   ethyl-4-methoxycinnamate | -4.911 | Terpenoids | (9) |
|  |   Asiatica acid | INACTIVE | Terpenoids | (10) |
|  |   Ent pimarane | -4.4200 | Terpenoids | (10) |
|  |   asiaticusinols A | INACTIVE | Terpenoids | (11) |
|  |   14′,15′-dihydroasiaticusin A methyl ester | -5.3095 | Terpenoids | (11) |
|  |   asiaticusinols B | -5.3940 | Terpenoids | (11) |
|  |   asiaticusinols C | INACTIVE | Terpenoids | (11) |
|  |   asiaticusin A | INACTIVE | Terpenoids | (11) |
|  |   asiachromenic acid | -6.2401 | Terpenoids | (11) |
|  |   Beta­-Elemene | -3.8614 | Terpenoids | (12) |
|  |   spathulenol | -5.7074 | Terpenoids | (12) |
|  |   Gracilin | -4.0798 | Terpenoids | (13) |
|  |   Ginsenosides Re | INACTIVE | Terpenoids | (13) |
|  |   Ginsenosides Rg1 | -7.2240 | Terpenoids | (13) |
|  |   Ginsenoisdes Rg3 | INACTIVE | Terpenoids | (13) |
|  |   Aperterpene B | -4.5958 | Terpenoids | (13) |
|  |   Asperterpene A | -4.7120 | Terpenoids | (13) |
|  |   Terreusterpene A | INACTIVE | Terpenoids | (14) |
|  |   Terreusterpenes B | INACTIVE | Terpenoids | (14) |
|  |   Terreusterpenes C | INACTIVE | Terpenoids | (14) |
|  |   Terreusterpenes D | INACTIVE | Terpenoids | (14) |
|  |   Spiroterreusnoids A | INACTIVE | Terpenoids | (15) |
|  |   Spiroterreusnoids B | INACTIVE | Terpenoids | (15) |
|  |   Spiroterreusnoids C | INACTIVE | Terpenoids | (15) |
|  |   Spiroterreusnoids D | INACTIVE | Terpenoids | (15) |
|  |   Spiroterreusnoids E | INACTIVE | Terpenoids | (15) |
|  |   Spiroterreusnoids F | INACTIVE | Terpenoids | (15) |
|  |   Phomophyllin A | INACTIVE | Terpenoids | (16) |
|  |   Phomophyllin B | INACTIVE | Terpenoids | (16) |
|  |   Phomophyllin C | INACTIVE | Terpenoids | (16) |
|  |   Phomophyllin D | INACTIVE | Terpenoids | (16) |
|  |   Phomophyllin E | INACTIVE | Terpenoids | (16) |
|  |   Phomophyllin F | INACTIVE | Terpenoids | (16) |
|  |   Phomophyllin G | INACTIVE | Terpenoids | (16) |
|  |   Phomophyllin I | INACTIVE | Terpenoids | (16) |
|  |   radulone B | INACTIVE | Terpenoids | (16) |
|  |   Onitin | -4.3457 | Terpenoids | (16) |
|  |   7-hydroxy-10-oxodehydrodihydrobotrydial | INACTIVE | Terpenoids | (16) |
|  |   Sargahydroquinoic acid | -6.0718 | Terpenoids | (16) |
|  |   Sargaquinoic acid | -5.5226 | Terpenoids | (16) |
|  |   sargachromenol | -7.5614 | Terpenoids | (16) |
|  |   hecogenin | -4.2036 | Terpenoids | (16) |
|  |   Cholest-4-en-3-one | -5.2137 | Terpenoids | (16) |
|  |   Asperterpene F | -5.7779 | Terpenoids | (16) |
|  |   Asperterpene E | INACTIVE | Terpenoids | (16) |
|  |   Asperterpene J | -6.5013 | Terpenoids | (16) |
|  |   Deadalol C | -5.1733 | Terpenoids | (16) |
|  |   21b,24-dihydroxyserrat-14-en-3a-yl acetate | INACTIVE | Terpenoids | (17) |
|  |   21a-hydroxyserrat-14-en-3b-yl acetate | INACTIVE | Terpenoids | (17) |
|  |   serrat-14-en-3b,21a-diol | -4.1319 | Terpenoids | (17) |
|  |   21b-hydroxyserrat-14-en-3b-yl acetate | INACTIVE | Terpenoids | (17) |
|  |   21b-hydroxyserrat-14-en-3a-yl acetate | INACTIVE | Terpenoids | (17) |
|  |   serrat-14-en-3b,21b-diol | INACTIVE | Terpenoids | (17) |
|  |   16-oxo-21b,24-dihydroxyserrat-14-en-3a-yl acetate | -4.7107 | Terpenoids | (17) |
|  |   3a,21b-dihydroxyserrat-14-en-16-one | -4.9911 | Terpenoids | (17) |
|  |   16-oxo-21b-hydroxyserrat-14-en-3a-yl acetate | -4.4931 | Terpenoids | (17) |
|  |   lycoclavanol | -4.7423 | Terpenoids | (17) |
|  |   Cholesta-4,6-dien-3-ol | -5.5885 | Terpenoids | (18) |
|  |   hurgadacin | -5.3928 | Terpenoids | (18) |
|  | Deoxyneocryptotashinone | -4.3981 | Terpenoids | (19) |
|  | Grandifolia F | -5.2855 | Terpenoids | (19) |
|  | Ferruginol | INACTIVE | Terpenoids | (19) |
|  | Cryptotanshinone | -3.0183 | Terpenoids | (19) |
|  | Tanshinone IIA | -3.2989 | Terpenoids | (19) |
|  | Tanshinol B | -3.4927 | Terpenoids | (19) |
|  | Tanshinone IIB | -4.5119 | Terpenoids | (19) |
|  | Tanshinonal | -4.3516 | Terpenoids | (19) |
|  | 15,16-Dihydrotanshinone I | -4.3911 | Terpenoids | (19) |
|  | Tanshinone I | -4.3729 | Terpenoids | (19) |
|  | Dehydroanshenol A | -5.4596 | Terpenoids | (19) |
|  | Succedaneaflavanone | -6.4854 | flavanoid | (20) |
|  | rhusflavanone | -6.1825 | flavanoid | (20) |
|  | rhusflavone | -5.7260 | flavanoid | (20) |
|  | agathisflavone | -5.8907 | flavanoid | (20) |
|  | cupressoflavone | -5.8345 | flavanoid | (20) |
|  | Messuaferone B | -5.8676 | flavanoid | (20) |
|  | Luteolin | -4.3348 | Flavonoid | (21) |
|  | Rosmarinic acid | -5.7961 | Flavonoid | (21) |
|  | sophoraflavanone G | -6.0268 | Flavonoid | (22) |
|  | kurarinone | -6.1985 | Flavonoid | (22) |
|  | leachianone | INACTIVE | Flavonoid | (22) |
|  | kushenol A | -5.0372 | Flavonoid | (22) |
|  | 2S-2-methoxy kurarinone | -6.6270 | Flavonoid | (22) |
|  | kushenol T | -5.7134 | Flavonoid | (22) |
|  | kurarinol | -5.7033 | Flavonoid | (23) |
|  | Allo-aloeresin D | 6.6180 | Flavonoid | (24) |
|  | Rebaichromone | -5.6286 | Flavonoid | (24) |
|  | Aloesin | -4.6579 | Flavonoid | (24) |
|  | 8-C-glucosyl-7-O-methylaloediol | -5.5429 | Flavonoid | (24) |
|  | 8-C-glucosyl-7-O-methoxy-R-aloesol | -5.4096 | Flavonoid | (24) |
|  | 8-C-glucosyl-R-alesol | -5.9343 | Flavonoid | (24) |
|  | S-aloesinol-9a | -5.9619 | Flavonoid | (24) |
|  | 2-O-coumaryl-S-aloesinol | -7.2554 | Flavonoid | (24) |
|  | 2-O-p-methoxy-E-cinnamoyl-S-aloesinol | INACTIVE | Flavonoid | (24) |
|  | 2-Feruloylaloesin | -6.4431 | Flavonoid | (24) |
|  | Aloeresin A | -6.0078 | Flavonoid | (24) |
|  | 2-feruloyl-7-O-methylaloesin | -6.5564 | Flavonoid | (24) |
|  | Aloeresin D | -5.8983 | Flavonoid | (24) |
| 1. E | Vistinol E | -5.8846 | Flavonoid | (25) |
|  | E viniferin | -5.7824 | Flavonoid | (25) |
|  | Ampelopsin A | -5.0040 | Flavonoid | (25) |
|  | Vitisin A | -5.4568 | Flavonoid | (25) |
|  | Vitsin B | INACTIVE | Flavonoid | (25) |
|  | urolithin A | -4.5906 | Flavonoid | (26) |
|  | Icarrin | -6,2565 | Flavonoid | (26) |
|  | hydroxytyrosol | INACTIVE | Flavonoid | (26) |
|  | Quericitin | -5.2068 | Flavonoid | (26) |
|  | Hypericin | -5.6830 | Flavonoid | (26) |
|  | Mangiferin | -5.2530 | Flavonoid | (26) |
|  | Epigallocatechin -3-gallate | -5.9085 | Flavonoid | (26) |
|  | Oleuropein | -6.1779 | Flavonoid | (26) |
|  | resveratrol | -4.3917 | Flavonoid | (26) |
|  | Apigenin | -4.2419 | Flavonoid | (27) |
|  | Taiwaniaflavone(apeginin dimer) | -5.8198 | Flavonoid | (27) |
|  | Epicatechin | -4.3628 | Flavonoid | (28) |
|  | Epigallocatechin | -4.7795 | Flavonoid | (28) |
|  | desmethylanhydroicaritin | INACTIVE | Flavonoid | (29) |
|  | 8-lavadulylkaempferol | -5.7264 | Flavonoid | (29) |
|  | kushenol C | -6.0315 | Flavonoid | (29) |
|  | xanthohumol | INACTIVE | Flavonoid | (29) |
|  | kuraridinol | -6.1089 | Flavonoid | (29) |
|  | leachianone G | -5.7450 | Flavonoid | (29) |
|  | 3beta,7,4-trihydroxy-5-methoxy-8(gamma,gamma-dimethyl-flavanone) | -5.4711 | Flavonoid | (29) |
|  | kushenol E | -5.8272 | Flavonoid | (29) |
|  | kuraridin | -5.2998 | Flavonoid | (29) |
|  | 2,3-dehydroamentoflavone | -6.2583 | Flavonoid | (30) |
|  | Robustaflavone | -6.1796 | Flavonoid | (30) |
|  | Cupressoflavone | -5.8345 | Flavonoid | (30) |
|  | Hinokiflavone | -6.9577 | Flavonoid | (30) |
|  | Norartocarpetin | -4.9285 | Flavonoid | (30) |
|  | kuwanon C | -5.3426 | Flavonoid | (30) |
|  | Morusin | -6.0746 | Flavonoid | (30) |
|  | Morusinol | -5.3941 | Flavonoid | (30) |
|  | Mormin | INACTIVE | Flavonoid | (30) |
|  | Neocyclomorusin | -5.1843 | Flavonoid | (30) |
|  | Kuwanon A | -5.3086 | Flavonoid | (30) |
|  | 1,4-naphthouinone derivative | INACTIVE | Flavonoid | (30) |
|  | Aloveroside A | -5.9357 | Flavonoid | (30) |
|  | 2,2,4-Trihydroxychalcone | -5.3705 | Flavonoid | (31) |
|  | Boeravinone T | -3.8158 | Flavonoid | (32) |
|  | Boeravinone U | -4.4189 | Flavonoid | (32) |
|  | Boeravinone J | -4.2121 | Flavonoid | (32) |
|  | Boeravinone B | -4.2716 | Flavonoid | (32) |
|  | Boeravinone X | -6.0454 | Flavonoid | (32) |
|  | Corilagin | -5.5514 | Flavonoid | (33) |
|  | geraniin | -7.8132 | Flavonoid | (33) |
|  | Boeravinone D | -4.9279 | Flavonoid | (34) |
|  | boeravinone A methyl ether | -5.6794 | Flavonoid | (34) |
|  | mirabijalone D | INACTIVE | Flavonoid | (34) |
|  | abronione A | -4.4802 | Flavonoid | (34) |
|  | neocoylin | -6.0114 | Flavonoid | (10) |
|  | sebestenoids C | -6.4564 | Flavonoid | (10) |
|  | myricetin | -4.6883 | Flavonoid | (10) |
|  | morin | -4.3255 | Flavonoid | (10) |
|  | Amentoflavaone | -5.7224 | Flavonoid | (16) |
|  | Sequoiaflavone | -6.6338 | Flavonoid | (16) |
|  | Biolobetin | -6.2286 | Flavonoid | (16) |
|  | Sotestuflavone | -7.0632 | Flavonoid | (16) |
|  | Podocarpusflavone A | -6.4916 | Flavonoid | (16) |
|  | Ginkgetin | -6.5080 | Flavonoid | (16) |
|  | Amentoflavone -7,7-dimethyl ether | -5.9767 | Flavonoid | (16) |
|  | Podocarpusflavone B | -6.7313 | Flavonoid | (16) |
|  | Isoginkgetin | -7.1784 | Flavonoid | (16) |
|  | 2,3-dihydro-6-methylginkgetin | -6.6668 | Flavonoid | (16) |
|  | cyclomorusin | -5.9901 | Flavonoid | (16) |
|  | 5,7,2,4-tetrahydroxy-8-(3,7-dimethyl-2,6-octadienyl)isoflavone | -5.9002 | Flavonoid | (16) |
|  | nobiletin | -5.7195 | Flavonoid | (16) |
|  | Tangeretin | -4.9332 | Flavonoid | (16) |
|  | Senesetin | -5.3528 | Flavonoid | (16) |
|  | (S)5,7,3,5-tetrahydroxy-dihydroflavone-7-O-(6-galloyl)-beta-D-glucopyranose | INACTIVE | Flavonoid | (16) |
|  | (S)5,7,3,5-tetrahydroxy-hydroflavanone-7-O-beta-D-glucopyranose | INACTIVE | Flavonoid | (16) |
|  | 4,2,6-trihydroxy-dihydrochalcone-4-O-(6-galloyl)-beta-D-glucopyranose | -6.3546 | Flavonoid | (16) |
|  | Phloretin 4-O-[4,6-O-(S)-HHDP]-beta-D-glucopyranose | -6.7232 | Flavonoid | (16) |
|  | 3,4,2,6-tetrahydroxydihydroflavone- 4-O-beta-D-glucopyranose | -5.1473 | Flavonoid | (16) |
|  | 3,4,2,6-tetrahydroxydihydroflavone-dihydrochalcone- 4-O-(6-galloyl)-beta-D-glucopyranose | -6.1821 | Flavonoid | (16) |
|  | quercetin3-O-glucoside | -5.5804 | Flavonoid | (16) |
|  | Vitexin | -5.6445 | Flavonoid | (16) |
|  | didymin | -6.3507 | Flavonoid | (16) |
|  | poncirin | -6.7743 | Flavonoid | (16) |
|  | prunin | -5.4357 | Flavonoid | (16) |
|  | 3,5,7,3,4-pentamethoxyflavone | -5.0086 | Flavonoid | (35) |
|  | Tacrine | INACTIVE | Flavonoid | (36) |
|  | QUD | -5.5786 | Flavonoid | (36) |
|  | donepezil | -5.0672 | Flavonoid | (36) |
|  | rubrofusarin | -4.1082 | Flavonoid | (36) |
|  | Rubrofusarin 6-O-beta-D-Glucopyranoside | -6.4718 | Flavonoid | (36) |
|  | Rubrofusarin 6-O-beta-D-gentiobioside | -6.7433 | Flavonoid | (36) |
|  | Nor-rubrofusarin 6-O-beta-D-Glucoside | -4.7626 | Flavonoid | (36) |
|  | Isorubrofusarin 10-O-beta-D-gentiobioside | -5.4240 | Flavonoid | (36) |
|  | E veniferin | -5.7824 | Flavonoid |  |
|  | cardamonin | -4.6777 | Flavonoid | (36) |
|  | pinocembrin | -3.8127 | Flavonoid | (36) |
|  | pinostrobin | -4.5994 | Flavonoid | (36) |
|  | naringenin | -4.2349 | Flavonoid | (37) |
|  | Biochanin A | -4.2468 | Flavonoid | (38) |
|  | ellagic acid | -4.5833 | Phenolic compounds | (39) |
|  | Punicalagin | -6.1224 | Phenolic compounds | (39) |
|  | Ferulic acid | -3.8634 | Phenolic compounds | (6) |
|  | ursolic acid | -4.6932 | Phenolic compounds | (40) |
|  | p- coumaric acid | -5.8607 | Phenolic compounds | (40) |
|  | gallic acid | INACTIVE | Phenolic compounds | (40) |
|  | oxyresveratrol | -5.0323 | Phenolic compounds | (41) |
|  | Cis-scirpusin A | -5.0227 | Phenolic compounds | (41) |
|  | Hispidin | -4.7963 | Phenolic compounds | (41) |
|  | 1,2,3,4,6,-Pentagalloyl-b-D-glucopyranoside | -7.1209 | Phenolic compounds | (41) |
|  | -epigallocatechin gallate | -4.6773 | Phenolic compounds | (41) |
|  | -epicatechin gallate | -6.7487 | Phenolic compounds | (41) |
|  | catechin positive r negative | -4.6741 | Phenolic compounds | (41) |
|  | Tellimagrandin II | -6.7608 | Phenolic compounds | (41) |
|  | Heparin | -6.1324 | Phenolic compounds | (41) |
| 1. L | XESTOSAPROL H | -4.9754 | Phenolic compounds |  |
|  | XESTOSAPROL F | -4.6571 | Phenolic compounds |  |
|  | neocorylin | -4.5169 | Phenolic compounds | (42) |
|  | bakuchiol | -5.0393 | Phenolic compounds | (42) |
|  | psoralen | INACTIVE | Phenolic compounds | (42) |
|  | vachromene | -5.2175 | Phenolic compounds | (42) |
|  | isobavachromene | -4.7652 | Phenolic compounds | (42) |
|  | 7,8-dihydro-8-(4-hydrophenyl)-2,2-dimethyl-2*H*,6*H*-[1,2-*b*:5,4-*b*′]dipyran-6-one | -5.5101 | Phenolic compounds | (42) |
|  | bavachinin | -5.4365 | Phenolic compounds | (42) |
|  | bavachalcone | -5.2996 | Phenolic compounds | (42) |
|  | isobavachalcone | -4.6263 | Phenolic compounds | (42) |
|  | Protocatechuic acid | -6.0210 | Phenolic compounds | (43) |
|  | Vanillic acid | ­­-6.2950 | Phenolic compounds | (43) |
|  | Syringic acid | -5.7523 | Phenolic compounds | (43) |
|  | Caffeic acid | -6.2029 | Phenolic compounds | (43) |
|  | Sinapinic acid | -6.4938 | Phenolic compounds | (43) |
|  | P-Hydroxy benzoic acid derivative | -6.5576 | Phenolic compounds | (43) |
|  | p-coumaric acid | -5.8607 | Phenolic compounds | (43) |
|  | 2-[(Z)-heptadec-11-enyl]-6-hydroxybenzoic acid | -5.7262 | Phenolic compounds | (16) |
|  | 2-[(6Z,9Z,12Z)-heptadeca-6,9,12-trienyl]-6-hydroxybenzoic acid | -5.5803 | Phenolic compounds | (16) |
|  | 2-[(9Z,12Z)-heptadeca-9,12-dienyl]-6-hydroxybenzoic acid | -7.5967 | Phenolic compounds | (16) |
|  | 2-hydroxy-6-(12-phenyldodecyl)-benzoic acid | -7.0708 | Phenolic compounds | (16) |
|  | 5-geranyloxy-8-methoxypsoralen | -5.3355 | Phenolic compounds | (16) |
|  | bergamottin | -5.2529 | Phenolic compounds | (16) |
|  | 8-geranyloxypsoralen | -5.9435 | Phenolic compounds | (16) |
|  | 8-geranyloxy-5-methoxypsoralen | -5.8578 | Phenolic compounds | (16) |
|  | Phellopterin | -5.5020 | Phenolic compounds | (16) |
|  | kinidilin | -5.1998 | Phenolic compounds | (16) |
|  | Taxifolin | -4.6154 | Phenolic compounds | (16) |
|  | 57,4-trihydroxy-2-styrylchromone | -5.1286 | Phenolic compounds | (16) |
|  | 9-methoxycalocedrin | -5.9871 | Phenolic compounds | (16) |
|  | Calocedrin | -5.3532 | Phenolic compounds | (16) |
|  | savinin | -5.1893 | Phenolic compounds | (16) |
|  | alpha-methyl artoflavanocoumarin | -4.7288 | Phenolic compounds | (16) |
|  | 10-oxowiddrol | INACTIVE | Phenolic compounds | (16) |
|  | (7S,8R)-dihydro-3-hydroxy-8-hydroxymethyl-7-(4-hydroxy-3-methoxyphenyl)-1-benzofuranpropanol | -4.5314 | Phenolic compounds | (16) |
|  | styraxlignolide C | -5.4897 | Phenolic compounds | (16) |
|  | 3,5,7-trihydroxy-4-methoxycoumarano-chroman-4-one | -4.1164 | Phenolic compounds | (16) |
|  | lupiwighteone | -5.6575 | Phenolic compounds | (16) |
|  | 6-formyl umbelliferone | INACTIVE | Phenolic compounds | (16) |
|  | 8-formyl umbelliferone | INACTIVE | Phenolic compounds | (16) |
|  | umbelliferone | INACTIVE | Phenolic compounds | (16) |
|  | gartanin | -5.4678 | Phenolic compounds | (16) |
|  | 8-O-deoxygartanin | -5.5265 | Phenolic compounds | (16) |
|  | graciniafuran | -5.6524 | Phenolic compounds | (16) |
|  | Gamma-mangostin | -5.5198 | Phenolic compounds | (16) |
|  | Garcinone C | -5.3602 | Phenolic compounds | (16) |
|  | Garcinone D | -5.9912 | Phenolic compounds | (16) |
|  | Gallocatechin gallate | INACTIVE | Phenolic compounds |  |
|  | veraphenol | -3.7630 | Phenolic compounds |  |
|  | 2,3,6-tribromo-4,5-dihydroxybenzyl alcohol | INACTIVE | Phenolic compounds | (16) |
|  | 2,3,6-tribromo-4,5-dihydroxybenzyl methylether | INACTIVE | Phenolic compounds | (16) |
|  | bis-(2,3,6-tribromo-4,5-dihydroxybenzyl) ether | -4.8103 | Phenolic compounds | (16) |
|  | 3,4-Di-o-caffeoylquinic acid | -5.2299 | Phenolic compounds | (44) |
|  | Scopoletin | INACTIVE | Phenolic compounds | (44) |
|  | 7-O-methylwogonin | -4.8110 | Phenolic compounds | (44) |
|  | onysilin | -4.6572 | Phenolic compounds | (44) |
|  | Transferulic acid | -3.8742 | Phenolic compounds | (44) |
|  | Beta-sitostenone | -4.8962 | Phenolic compounds | (44) |
|  | isoimperatorin | -4.5774 | Phenolic compounds | (45) |
|  | imperatorin | -4.9714 | Phenolic compounds | (45) |
| 1. g | (+)-byakangelicol | INACTIVE | Phenolic compounds | (45) |
|  | (+)-byakangelicin | -5.3278 | Phenolic compounds | (45) |
|  | (+)-Oxypeucedanin | -4.6903 | Phenolic compounds | (45) |
|  | Phlorofurofeukoeckol-A | -6.6132 | Polyphenol  Xanthones | (16) |
|  | 6,6-bieckol | -7.0295 | Phenolic compounds | (16) |
|  | dieckol | -7.4846 | Phenolic compounds | (46) |
|  | eckol | -5.5784 | Phenolic compounds | (46) |
|  | 8,8-bieckol | -7.0073 | Phenolic compounds | (46) |
|  | bavachin | -5.0081 | Phenolic compounds | (47) |
|  | Salvianolic acid A | -6.0858 | Phenolic compounds | (19) |
|  | Salvianolic acid B | -6.4199 | Phenolic compounds | (19) |
|  | Salvianolic acid C | -6.4398 | Phenolic compounds | (19) |
|  | Magnesium lithospermate | Inactive | Phenolic compounds | (19) |
|  | sesamin | -4.7439 | Polyphenol  lignans | (48) |
|  | Sesamonil | -4.6086 | Polyphenol  lignans | (48) |
|  | -7a,8a cis E veniferin | -6.5748 | stilbenes | (49) |
|  | trans E viniferin | -6.1595 | stilbenes | (49) |
|  | cis-E-viniferin | -6.0086 | stilbenes | (49) |
|  | gentin H | -7.1600 | stilbenes | (49) |
|  | vitisinol C | -5.8406 | stilbenes | (49) |
|  | suffruticosol A | -6.1759 | stilbenes | (49) |
|  | suffruticosol B | -5.6547 | stilbenes | (49) |
|  | Dendrobine | INACTIVE | Alkaloids | (50) |
|  | dendrobine N oxide | -3.1444 | Alkaloids | (50) |
|  | nobilonine | -4.3156 | Alkaloids | (50) |
|  | 6-Hydroxy-nobilonine | -4.0603 | Alkaloids | (50) |
|  | Dendroxine | INACTIVE | Alkaloids | (50) |
|  | 13-Hydroxy-14-oxodendrobine | INACTIVE | Alkaloids | (50) |
|  | Berberine | -4.9597 | Alkaloids | (51) |
|  | Magnoflorine | -4.5579 | Alkaloids | (51) |
|  | Jateorrhizine | -5.9985 | Alkaloids | (51) |
|  | Coptisine | -4.0416 | Alkaloids | (51) |
|  | palmatine | -5.0657 | Alkaloids | (51) |
|  | Epiberberine | -5.8654 | Alkaloids | (51) |
|  | Groenlandicine | -4.5313 | Alkaloids | (51) |
|  | Dictazole A | -5.6967 | Alkaloids | (10) |
|  | bastadin 9 | -6.1574 | Alkaloids | (10) |
|  | Northalifoline | INACTIVE | Alkaloids | (16) |
|  | liensinine | -6.4458 | Alkaloids | (16) |
|  | Neferine | -7.3946 | Alkaloids | (16) |
|  | 31-hydroxybuxatrienone | -4.5302 | Alkaloids | (16) |
|  | Nb-demethylpapillotrienine | -4.5034 | Alkaloids | (16) |
|  | (+)-canadine |  | Alkaloids | (52) |
|  | (+)-corydaline | -5.5990 | Alkaloids | (52) |
|  | (+)-tetrahydropalmatine | -4.8874 | Alkaloids | (52) |
|  | (-)-isocorypalmine | -5.7624 | Alkaloids | (52) |
|  | (−)-scoulerine | -4.5648 | Alkaloids | (52) |
|  | (-)-corycavamine | -5.8616 | Alkaloids | (52) |
|  | (+)-corynoline | INACTIVE | Alkaloids | (52) |
|  | (±)-corycavidine | INACTIVE | Alkaloids | (52) |
|  | allocryptopine | INACTIVE | Alkaloids | (52) |
|  | (+)-corynoline | INACTIVE | Alkaloids | (52) |
|  | (+)-corydine | -5.6713 | Alkaloids | (52) |
|  | (+)-bulbocapnine | -5.9955 | Alkaloids | (52) |
|  | (-)-sinoacutine | INACTIVE | Alkaloids | (52) |
|  | (+)-N-methyllaurotetanine | -5.6497 | Alkaloids | (52) |
|  | Physicon | -4.4198 | anthraquinone | (53) |
|  | obtusifolin | -4.8635 | anthraquinone | (53) |
|  | obtusin | -4.7612 | anthraquinone | (53) |
|  | Aurantio-Obtusin | -4.2245 | anthraquinone | (53) |
|  | Chryso-obtusin | -5.2705 | anthraquinone | (53) |
|  | emodin | -3.6792 | anthraquinone | (53) |
|  | alaternin |  | anthraquinone | (53) |
|  | questin | -4.3366 | anthraquinone | (53) |
|  | 2-Hydroxy emodin 1-methyl ether | -4.2606 | anthraquinone | (52 |
|  | Aloe-emodin | -4.2715 | anthraquinone | (52 |
|  | gluco-aurantio obtusin | -5.8694 | anthraquinone | (53) |
|  | Gluco-obtusifolin | -6.2670 | anthraquinone | (53) |
|  | Chryso-obtusin-2-O-beta-D-glucoside | -4.9633 | anthraquinone | (53) |
|  | cassitoroside | -6.2223 | anthraquinone | (53) |
|  | Toralactone gentiobioside | -5.7024 | anthraquinone | (53) |
|  | cassiaside | -5.2294 | anthraquinone | (53) |
|  | Chrysophanol tetraglucoside | -7.4000 | anthraquinone | (53) |
|  | Chrysophanol triglucoside |  | anthraquinone | (53) |
|  | alizarin-1-methylether | -4.1517 | anthraquinone | (16) |
|  | 1,2-dimethoxy-3-hydroxy anthraquinone | -4.7245 | anthraquinone | (16) |
|  | 2-methoxy anthraquinone | -4.0045 | anthraquinone | (16) |
|  | Rubiadin -1-methylether | -4.1021 | anthraquinone | (16) |
|  | 1-hydroxy-3-hydroxymethyl anthraquinone | -4.0383 | anthraquinone | (16) |
|  | rubiadin |  | anthraquinone | (16) |
|  | Tellimagradin I | -8.1479 | tannins | (54)tannins |
|  | Isoterchebin |  | tannins | (54) |
|  | 1,2,3,6-Tetra-O-galloyl-beta-D-glucose | -7.5729 | tannins | (54) |
|  | 1,2,3,-Tri-O-galloyl-beta-D-glucose | -6.1776 | tannins | (54) |
|  | melberrofuran G | -8.3279 |  | (16) |
|  | Albanol B | -6.5116 |  | (16) |
|  | Kuwanon G | -6.0262 |  | (16) |
|  | Moracin M | -4.0918 |  | (55) |
|  | Moracin O | -4.1480 |  | (55) |
|  | Moracin P | -5.0252 |  | (55) |
|  | Moracin S | -5.3319 |  | (55) |
|  | glycyrrhizin | INACTIVE | glycosides | (56) |
|  | 18beta-glycyrrhetinic acid | INACTIVE | glycosides | (56) |
|  | 18alpha-glycyrrhetinic acid | -4.4217 | glycosides | (56) |
|  | (8E)-ligstroside | INACTIVE |  | (57) |
|  | bisdemethoxycurcumin | INACTIVE |  | (4) |
|  | curcumin | INACTIVE |  | (4) |
|  | demethoxycurcumin | INACTIVE |  | (4) |
|  | Beta bisaboline | INACTIVE |  | (4) |
|  | ISOXANTHOHUMOL | INACTIVE |  | (55) |
|  | AR-sesquiphellandrene | INACTIVE |  | (55) |
|  | BETA PHOMOPHYLLIN D | INACTIVE |  | (55) |
|  | Sebestenoids D | -7.2018 |  | (10) |

**References**

1. Choi YH, Choi CW, Kim JK, Jeong W, Park GH, Hong SS. (‒)-Pteroside N and pterosinone, new BACE1 and cholinesterase inhibitors from Pteridium aquilinum. Phytochemistry Letters. 2018 Oct 1;27:63–8.

2. Matsumura S, Murata K, Zaima N, Yoshioka Y, Morimoto M, Kugo H, et al. Inhibitory Activities of Essential Oil Obtained from Turmeric and Its Constituents against β-Secretase.

3. Youn K, Jeong WS, Jun M. β-secretase (BACE1) inhibitory property of loganin isolated from Corni fructus. Natural Product Research. 2013;27(16):1471–4.

4. Murata K, Matsumura S, Yoshioka Y, Ueno Y, Matsuda H. Screening of β-secretase and acetylcholinesterase inhibitors from plant resources. Journal of Natural Medicines. 2014 Sep 25;69(1):123–9.

5. Jana CK, Hoecker J, Woods TM, Jessen HJ, Neuburger M, Gademann K. Synthesis of Withanolide A, Biological Evaluation of Its Neuritogenic Properties, and Studies on Secretase Inhibition. Angewandte Chemie International Edition. 2011 Aug 29;50(36):8407–11.

6. Rauf A, Uddin G, Khan A, Siddiqui BS, Arfan M, Dalvandi K, et al. Pistagremic acid, a novel β-secretase enzyme (BACE1) inhibitor from Pistacia integerrima Stewart. Natural Product Research. 2015 Sep 17;29(18):1735–8.

7. Marumoto S, Okuno Y, Miyazawa M. Inhibition of β-secretase activity by monoterpenes, sesquiterpenes, and C13 norisoprenoids. Journal of Oleo Science. 2017;66(8):851–5.

8. Jung HA, Lee EJ, Kim JS, Kang SS, Lee JH, Min BS, et al. Cholinesterase and BACE1 inhibitory diterpenoids from Aralia cordata. Archives of Pharmacal Research. 2009 Oct;32(10):1399–408.

9. Matsumura S, Murata K, Yoshioka Y, Matsuda H. Search for β-Secretase Inhibitors from Natural Spices. Natural Product Communications. 2016;11(4):507–10.

10. Williams P, Sorribas A, Howes MJR. Natural products as a source of Alzheimer’s drug leads. Vol. 28, Natural Product Reports. 2011. p. 48–77.

11. Yatsu G, Kino Y, Sasaki H, Satoh JI, Kinoshita K, Koyama K. Meroterpenoids with BACE1 Inhibitory Activity from the Fruiting Body of Boletinus asiaticus. Journal of Natural Products. 2019 Jul 26;82(7):1797–801.

12. Kawamoto H, Takeshita F, Murata K. Inhibitory Effects of Essential Oil Extracts From Panax ginseng Against β-Secretase and Cholinesterases . Natural Product Communications. 2019 Aug;14(8):1934578X1987344.

13. Naushad M, Durairajan SSK, Bera AK, Senapati S, Li M. Natural Compounds with Anti-BACE1 Activity as Promising Therapeutic Drugs for Treating Alzheimer’s Disease. Vol. 85, Planta Medica. Georg Thieme Verlag; 2019. p. 1316–25.

14. Qi C, Qiao Y, Gao W, Liu M, Zhou Q, Chen C, et al. New 3,5-dimethylorsellinic acid-based meroterpenoids with BACE1 and AchE inhibitory activities from Aspergillus terreus. Organic and Biomolecular Chemistry. 2018;16(46):9046–52.

15. Qi C, Zhou Q, Gao W, Liu M, Chen C, Li XN, et al. Anti-BACE1 and anti-AchE activities of undescribed spiro-dioxolane-containing meroterpenoids from the endophytic fungus Aspergillus terreus Thom. Phytochemistry. 2019 Sep 1;165.

16. Murata K. Chemical Diversity of β-Secretase Inhibitors From Natural Resources. Nat Prod Commun. 2019 Dec 1;14(12):1934578X1989481.

17. Nguyen VT, Zhao BT, Seong SH, Kim JA, Woo MH, Choi JS, et al. Inhibitory effects of serratene-type triterpenoids from Lycopodium complanatum on cholinesterases and β-secretase 1. Chemico-Biological Interactions. 2017 Aug 25;274:150–7.

18. Zhu YZ, Liu JW, Wang X, Jeong IH, Ahn YJ, Zhang CJ. Anti-BACE1 and antimicrobial activities of steroidal compounds isolated from marine urechis unicinctus. Marine Drugs. 2018;16(3).

19. Yu T, Paudel P, Seong SH, Kim JA, Jung HA, Choi JS. Computational insights into β-site amyloid precursor protein enzyme 1 (BACE1) inhibition by tanshinones and salvianolic acids from Salvia miltiorrhiza via molecular docking simulations. Computational Biology and Chemistry. 2018 Jun 1;74:273–85.

20. Shrestha S, Park JH, Lee DY, Cho JG, Seo WD, Kang HC, et al. Cytotoxic and neuroprotective biflavonoids from the fruit of Rhus parviflora. Journal of the Korean Society for Applied Biological Chemistry. 2012 Aug;55(4):557–62.

21. Choi SH, Hur JM, Yang EJ, Jun M, Park HJ, Lee KB, et al. β-secretase (BACE1) inhibitors from Perilla frutescens var. acuta. Archives of Pharmacal Research. 2008 Feb;31(2):183–7.

22. Hwang EM, Ryu YB, Kim HY, Kim DG, Hong SG, Lee JH, et al. BACE1 inhibitory effects of lavandulyl flavanones from Sophora flavescens. Bioorganic and Medicinal Chemistry. 2008 Jul 15;16(14):6669–74.

23. Hwang EM, Ryu YB, Kim HY, Kim DG, Hong SG, Lee JH, et al. BACE1 inhibitory effects of lavandulyl flavanones from Sophora flavescens. Bioorganic and Medicinal Chemistry. 2008 Jul 15;16(14):6669–74.

24. Lv L, Yang QY, Zhao Y, Yao CS, Sun Y, Yang EJ, et al. BACE1 (β-secretase) inhibitory chromone glycosides from Aloe vera and Aloe nobilis. Planta Medica. 2008 Apr;74(5):540–5.

25. Yeon HC, Mi YY, Chun WC, Cha MR, Gyu HY, Dae YK, et al. A new specific BACE-1 inhibitor from the stembark extract of Vitis vinifera. Planta Medica. 2009 Apr;75(5):537–40.

26. Dhakal S, Kushairi N, Phan CW, Adhikari B, Sabaratnam V, Macreadie I. Dietary polyphenols: A multifactorial strategy to target alzheimer’s disease. Vol. 20, International Journal of Molecular Sciences. MDPI AG; 2019.

27. Thapa A, Chi EY. Biflavonoids as potential small molecule therapeutics for alzheimer’s disease. Advances in Experimental Medicine and Biology. 2015;863:55–77.

28. Cox CJ, Choudhry F, Peacey E, Perkinton MS, Richardson JC, Howlett DR, et al. Dietary (-)-epicatechin as a potent inhibitor of βγ-secretase amyloid precursor protein processing. Neurobiology of Aging. 2015 Jan 1;36(1):178–87.

29. Jung HA, Yokozawa T, Kim BW, Jung JH, Choi JS. Selective inhibition of prenylated flavonoids from Sophora flavescens against BACE1 and cholinesterases. American Journal of Chinese Medicine. 2010;38(2):415–29.

30. Abdul B, Vidhyavathi R, Magesh J, Vijayakumar M, Mustafa M, Marikar F. Synthesis and Development of BACE 1 Inhibitor for Alzheimer’s Diseases from Medicinal Plants – Review Article. Annual Research & Review in Biology. 2018 Aug 18;28(3):1–18.

31. Zhu Z, Li C, Wang X, Yang Z, Chen J, Hu L, et al. 2,2’,4’-Trihydroxychalcone from Glycyrrhiza glabra as a new specific BACE1 inhibitor efficiently ameliorates memory impairment in mice. Journal of Neurochemistry. 2010 Jul;114(2):374–85.

32. Yang EJ, Lee T, Song KS. β-Secretase inhibition by *C* -methylisoflavones from *Abronia nana*. Natural Product Research. 2019 Jun 18;33(12):1705–12.

33. Youn K, Jun M. In vitro BACE1 inhibitory activity of geraniin and corilagin from geranium thunbergii. Planta Med. 2013;79(12):1038–42.

34. Yang EJ, Lee T, Song KS. β-Secretase inhibition by C-methylisoflavones from Abronia nana. Natural Product Research. 2018 Feb 1;1–8.

35. Shrestha S, Seong SH, Paudel P, Jung HA, Choi JS. Structure related inhibition of enzyme systems in cholinesterases and BACE1 in vitro by naturally occurring naphthopyrone and its glycosides isolated from cassia obtusifolia. Molecules. 2018;23(1).

36. Youn K, Jun M. Biological evaluation and docking analysis of potent BACE1 inhibitors from boesenbergia rotunda. Nutrients. 2019 Mar 1;11(3).

37. Ali MY, Jannat S, Edraki N, Das S, Chang WK, Kim HC, et al. Flavanone glycosides inhibit β-site amyloid precursor protein cleaving enzyme 1 and cholinesterase and reduce Aβ aggregation in the amyloidogenic pathway. Chemico-Biological Interactions. 2019 Aug 25;309.

38. Youn K, Park JH, Lee J, Jeong WS, Ho CT, Jun M. The identification of biochanin a as a potent and selective β-site app-cleaving enzyme 1 (Bace1) inhibitor. Nutrients. 2016 Oct 1;8(10).

39. Kwak HM, Jeon SY, Sohng BH, Kim JG, Lee JM, Lee KB, et al. beta-Secretase (BACE1) inhibitors from pomegranate (Punica granatum) husk. Archives of pharmacal research. 2005 Dec;28(12):1328–32.

40. Youn K, Jun M. Inhibitory Effects of Key Compounds Isolated from *Corni fructus* on BACE1 Activity. Phytotherapy Research. 2012 Nov;26(11):1714–8.

41. Sheean P, Rout MK, Head RJ, Bennett LE. Modulation of in vitro activity of zymogenic and mature recombinant human β-secretase by dietary plants. FEBS Journal. 2012 Apr;279(7):1291–305.

42. Choi YH, Yon CH, Hong KS, Yoo DS, Choi CW, Park WK, et al. In vitro BACE-1 inhibitory phenolic components from the seeds of Psoralea corylifolia. Planta Medica. 2008 Sep;74(11):1405–8.

43. Eom TK, Ryu BM, Lee JK, Byun HG, Park SJ, Kim SK. β-secretase inhibitory activity of phenolic acid conjugated chitooligosaccharides. Journal of Enzyme Inhibition and Medicinal Chemistry. 2013 Feb;28(1):214–7.

44. Panche AN, Chandra S, Diwan AD. Multi-target β-protease inhibitors from andrographis paniculata: In silico and in vitro studies. Plants. 2019 Jul 1;8(7).

45. Marumoto S, Miyazawa M. Î^2^-secretase inhibitory effects of furanocoumarins from the root of *Angelica dahurica*. Phytotherapy Research. 2009;n/a-n/a.

46. Lee J, Jun M. Dual BACE1 and cholinesterase inhibitory effects of phlorotannins from ecklonia cava-an in vitro and in silico study. Mar Drugs. 2019 Feb 1;17(2).

47. Xu QX, Hu Y, Li GY, Xu W, Zhang YT, Yang XW. Multi-target anti-Alzheimer activities of four prenylated compounds from Psoralea Fructus. Molecules. 2018;23(3).

48. Matsumura S, Murata K, Zaima N, Yoshioka Y, Morimoto M, Matsuda H, et al. Inhibitory Activities of Sesame Seed Extract and its Constituents against β-Secretase.

49. Choi CW, Choi YH, Cha MR, Kim YS, Yon GH, Hong KS, et al. In vitro BACE-1 inhibitory activity of resveratrol oligomers from the seed extract of paeonia lactiflora. Vol. 77, Planta Medica. 2011. p. 374–6.

50. Huang J, Huang N, Zhang M, Nie J, Xu Y, Wu Q, et al. Dendrobium alkaloids decrease Aβ by regulating α- And β-secretases in hippocampal neurons of SD rats. PeerJ. 2019;2019(9).

51. Anti-Alzheimer and Antioxidant Activities of Coptidis Rhizoma Alkaloids - Google Search [Internet]. [cited 2020 Jan 23]. Available from: https://www.google.com/search?q=Anti-Alzheimer+and+Antioxidant+Activities+of+Coptidis+Rhizoma+Alkaloids&rlz=1C1GCEA_enPK868PK868&oq=Anti-Alzheimer+and+Antioxidant+Activities+of+Coptidis+Rhizoma+Alkaloids&aqs=chrome..69i57j69i61.1006j0j7&sourceid=chrome&ie=UTF-8

52. Chlebek J, De Simone A, Hoš͗álková A, Opletal L, Pérez C, Pérez DI, et al. Application of BACE1 immobilized enzyme reactor for the characterization of multifunctional alkaloids from Corydalis cava (Fumariaceae) as Alzheimer’s disease targets. Fitoterapia. 2016 Mar 1;109:241–7.

53. Jung HA, Ali MY, Jung HJ, Jeong HO, Chung HY, Choi JS. Inhibitory activities of major anthraquinones and other constituents from Cassia obtusifolia against β-secretase and cholinesterases. J Ethnopharmacol. 2016 Sep 15;191:152–60.

54. Bhakta HK, Park CH, Yokozawa T, Tanaka T, Jung HA, Choi JS. Potential anti-cholinesterase and β-site amyloid precursor protein cleaving enzyme 1 inhibitory activities of cornuside and gallotannins from Cornus officinalis fruits. Arch Pharm Res. 2017 Jul 1;40(7):836–53.

55. Seong SH, Ha MT, Min BS, Jung HA, Choi JS. Moracin derivatives from Morus Radix as dual BACE1 and cholinesterase inhibitors with antioxidant and anti-glycation capacities. Life Sciences. 2018 Oct 1;210:20–8.

56. Wagle A, Seong SH, Zhao BT, Woo MH, Jung HA, Choi JS. Comparative study of selective in vitro and in silico BACE1 inhibitory potential of glycyrrhizin together with its metabolites, 18α- and 18β-glycyrrhetinic acid, isolated from Hizikia fusiformis. Archives of Pharmacal Research. 2018 Apr 1;41(4):409–18.

57. Matsumura S, Murata K, Yoshioka Y, Matsuda H. Search for β-Secretase Inhibitors from Natural Spices. Natural Product Communications. 2016;11(4):507–10.
