# Supplementary material for: Cheminformatics-driven discovery of natural isoquinoline alkaloid inhibitors of Beta-secretase I for Alzheimer’s management
Source: PLoS One. 2026 Mar 2;21(3):e0343717. doi: 10.1371/journal.pone.0343717 (PMC12952630; doi:10.1371/journal.pone.0343717)
Supplement: S2 Table — (DOCX) [file pone.0343717.s002.docx]

**Table S2.** Table shows docking results, interacting residues of the receptor protein, nature, structure and names of the top 50 scoring compounds.

| **Sr. No.** | **Compound Structure and Name** | **Docking Score** | **Chemical Nature/Class** | **Interacting residues of BACE 1** | **Reference** |
| --- | --- | --- | --- | --- | --- |
|  |   melberrofuran G | -8.3279 |  | (19) Gly173, Asn172, Gln73, Gln224, Val370, Asn223, Lys70, Pro369, Glu400, Ala218, GLy219, Phe220, Ser71, Leu222, Arg368, Glu371, Gly72, Pro221, Asn223 | (1) |
|  |   Tellimagradin I | -8.1479 | Tannins | (23) Thr375, Asp372, Glu371, Lys382, Val397, Ser71, Ala218, Pro221, Asn223, Gly219, Phe220, Pro369, Gly217, Val370, Lys70, Gly72, Glu400, Leu222, Arg368, Gln73, Gln224, Gly173, Asn172 | (2) |
|  |  geraniin | -7.8132 | Flavonoid | (21) Gln134, Gly291, Thr292, Ile171, Thr293, Ser71, Lys382, Pro369, Arg368, Val373, Val370, Glu371, Asp372, Leu328, Lys70, Pro221, Gln73, Gly72, Gln224, Leu222,Thr293 | (3) |
|  |   2-[(9Z,12Z)-heptadeca-9,12-dienyl]-6-hydroxybenzoic acid | -7.5967 | Phenolic compounds | (17) GlnB73, GlyB72, AsnB172, ileB171, LysB168, TyrA283, ProB105, HisB106, PheB108, ProB107, LysA300, GluA303, LysA299, SerB166, ThrB164, GluB165,PheB170 | (1) |
|  |   1,2,3,6-Tetra-O-galloyl-beta-D-glucose | -7.5729 | Tannins | (25) Gly325, Leu324, Leu328, Lys382, Glu371, Asp372, Val373, Arg368, Val370, Pro369, Asn294, Gly291, Thr292, Ile171, Ser386, Thr293, Gln73, Gly74, Asn223, Lys70, Pro221, | (2) |
|  |   sargachromenol | -7.5614 | Terpenoids  meroterpenoid | (20) Pro221, Leu222, Glu400, Phe220, Gly219, Gly217, Ala218, Val422, Gln364, Leu367, Ser71, Arg368, Glu72, Gln73, Pro369, Lys70,Val370, Glu371, Asp372, Val373 | (1) |
|  |   dieckol | -7.4846 | Phenolic compounds | (17) Thr375,Lys70, Gly173, Gln73, Val370, Asp372, Arg368, Lys382, Val373, Leu328, Gln327, Glu326, Pro221, Glu371, Gln224, Leu222, Asn223 | (4) |
|  |   Chrysophanol tetraglucoside | -7.4000 | anthraquinone | (23) Thr293, Leu324, Asn294, Ser386, Gly325, Glu326, Lys382, Thr133, Asp372, Arg368, Pro369, Glu371, Val370, Gly72, Ile171, Lys70, Leu222, Gln73, Asn172, Asn223, Gly173, Gln224 | (5) |
|  |   Neferine | -7.3946 | Alkaloids | (16) Thr375, Val373, Asp372, Glu371, Asn223, Val370. Gly173, Leu222, Gln73, Ser71, Gly72, Arg368, Pro221,Gln224, Lys70, Pro369 | (1) |
|  |   2-O-coumaryl-S-aloesinol | -7.2554 | Flavonoid | (19) Leu91,Asp289, Asp93, Gly291, Thr292, Ile179, Thr292, Ile187, Tyr259, Gly95, Arg189, Pro131, Ile287, Val393, Thr390, Arg296, Thr133, Tyr132, Gln134, Phe169 | (6) |
|  |   Ginsenosides Rg1 | -7.2240 | Terpenoids | (12) Glu371, Gln224, Lys70, Thr375, Val370, Asn223, Leu222, Pro369, Pro221, Gln73, Arg368, Gly72 | (7) |
|  |   Sebestenoids D | -7.2018 |  | (19) Ile171, Gly72, Val227, Gln224, Gly173,Pro221, Asn223, Leu222, Lys70, Gln73, Glu371, Pro369, Val370, Lys382, Arg368, Gly325, Glu326, Leu324, Asn294 | (8) |
|  | Isoginkgetin | -7.1784 | Flavonoid | (13) Thr375, Val373, Asp372, Glu371, Ile171, Asn223, Gly72, Gly173, Asn172, Leu222, Lys70, Gln224, Gln73, Arg368, Val370, Pro221, Pro369, Phe220 | (1) |
|  |   gentin H | -7.1600 | Stilbenes | (18) Val373, Val370, Lys382, Asp372, Leu328, Pro369, Pro221, Glu371, Asn223, Gln224, Gly72, Gln73, Arg368, Phe170, Lys168, Gly173, Asn172, Ile171 | (9) |
|  |   1,2,3,4,6,-Pentagalloyl-b-D-glucopyranoside | -7.1209 | Phenolic compounds | (23) Glu326, Gly325, Arg368, Lys382, Thr293, Thr292, Gly291, Gln134, Lys168, Gly72, Ile171, Asn172, Lys70, Leu222, Gly173, Asn223, Gln224, Ser71, Val373, Leu328, Asp382, Glu371, Gln73 | (10) |
|  |   2-hydroxy-6-(12-phenyldodecyl)-benzoic acid | -7.0708 | Phenolic compounds | (18) Leu222, Phe220, Gly219, Gly217, Ala218, Glu400, Gln364, Pro369, Ser71, Pro221, Glu371, Arg368, Lys70, Val370, Gln73, Gln224, Gly173, Asn172 | (1) |
|  | Sotestuflavone | -7.0632 | Flavonoid | (16) Ile171, Asn172, Gln224, Gly173, Gln73, Asn223, , Lys70, Leu222, Gly72, Pro369, Val370, Arg368, , Asp372, Glu371, Lys382, Leu328 | (1) |
|  |   6,6-bieckol | -7.0295 | Phenolic compounds | (15) Phe108, Lys168, Gln134, Gly72, Gln73, Lys70, Pro369, Glu371, Val370, Arg368, Gly325, Lys382, Phe170, Ile171, Asn172 | (1) |
|  |   8,8-bieckol | -7.0073 | Phenolic compounds | (13) Asp378, Ser376, Asp372, Val370, Glu371, Gln377, Glu371, Pro369, Leu222, Lys70, Gln224, Asn223, Gln73 | (4) |
|  |   Hinokiflavone | -6.9577 | Flavonoid | (15) Gly219, Pro221, Phe220, Ala218, Pro369, Gly217, Glu400,Ser71, Leu222, Val370, Arg368, Gly72, Lys70, Glu371, Gln73, | (11) |
|  |   poncirin | -6.7743 | Flavonoid | (15) Val373, Asp372, Val370, Glu371 Pro368, Asn223, Lys382, Arg368, Leu328, Pro221, Gly72, Gln73, Gln224, Lys70, Leu222 | (1) |
|  |   Tellimagrandin II | -6.7608 | Phenolic compounds | (18) Gln224, Asn223, Leu222, Gln73, Asn172, Gly72, Pro221, Ile171, Glu371, Lys382, Arg368, Glu326, Asp372, Val373, Gln327, Leu328, Val370, Lys70, | (10) |
|  |   -epicatechin gallate | -6.7487 | Phenolic compounds |  | (10) |
|  |   Rubrofusarin 6-O-beta-D-gentiobioside | -6.7433 | Flavonoid | (15) Glu326, Lys382, Leu328, Arg368, Val370, Val373, Asp372, Glu371, Asn223, Gln224, Gly173, Asn172, Gln73, Gly72, Lys70 | (12) |
|  | Podocarpusflavone B | -6.7313 | Flavonoid | (16) Thr376, Val373, Asp372, Ile171, Gln224, Gln371, Asp172, Gln73, Lys70, Leu222, Val370, Phe220, Arg368, Gly72, Pro221, Pro369 | (1) |
|  |   Phloretin 4-O-[4,6-O-(S)-HHDP]-beta-D-glucopyranose | -6.7232 | Flavonoid | (14) Lys168, Phe108, Phe170, Asn172, Ile171, Glu371, Vla370, Pro369, Pro221, Gln73, Leu222, Lys70, Gly72,Arg368 | (1) |
|  |   2,3-dihydro-6-methylginkgetin | -6.6668 | Flavonoid | (12) AsnB172, ProB107, PheB108, LysA199, HisB106, PheB170, LysB168, TyrA445, GluA280, TyrA283, SerA389, AspA284, | (1) |
|  |   Phlorofurofeukoeckol-A | -6.6132 | Polyphenol  Xanthones | (8) Arg255, Glu441 , Ile263, Thr353, Gly350, Glu351, Met349, Pro253, | (1) |
|  | Sequoiaflavone | -6.6338 | Flavonoid | (19) ThrB390, ValB393, ArgB296, TyrB259, IleB187, GlyB95, SerB96, ValB130, ThrB133, GlyB135, TyrB132, ProB131, TyrB129, GlnB134, LysB136, LeuA274, LysA275, LysB168, AspB167 | (1) |
|  |   2S-2-methoxy kurarinone | -6.6270 | Flavonoid | (15) Gln224, Ile171, Pro369, Lys382, Arg368, Gly72, Asn223, Gly173, Pro221, Asn172, Val370, Gln73, Lys70, Leu222, Glu371 | (13) |
|  |   -7a,8a cis E veniferin | -6.5748 | Stilbenes | (13) Lys382, Val370, Gln73, Gln224, Lys70, Arg368, Leu222, Pro369, Glu371, Gly72, Asn172, Asn223, Pro221, | (9) |
|  | 2-feruloyl-7-O-methylaloesin | -6.5564 | Flavonoid | (14) Asn 172, Gln224, Pro221, Asn223, Gln73, Lys70, Gly72, Arg368, Glu371, Asp372, Thr375, Asp372, Val370, Val373, | (6) |
|  |   P-Hydroxy benzoic acid derivative | -6.5576 | Phenolic compounds | (16) Phe220, Pro221, Pro369, Val370, Gly72, Arg368, Thr293, Gln134, Ile171, Asn172, Glu371, Gln73, Lys70, Leu222, Gln224, Asn223 | (14) |
|  |   Albanol B | -6.5116 |  | (15) Asn172, Gln224, Gly173, Gln73, Ile171, Lys168, Gln134, Gly72, Lys70, Leu222, Pro369, Pro221, Val370, Asn223, Glu371, | (1) |
|  |   Asperterpene J | -6.5013 | Terpenoids | (12) Thr390,Thr133, Lys285, Arg296, Val393, Tyr259, Trp258, Ile187, Val130, Pro131, Glu186, Arg189, | (1) |
|  |  Ginkgetin | -6.5080 | Flavonoid | (18) ValB393, BArg296, ThrB390, TyrB259, IleB187, GlyB95, ThrB133, proB131, ValB130, TyrB129, SerB96, LysA275, LeuA274, GlyB135, LysB136, AspB167, LysB168, TyrB132, | (1) |
|  |   Sinapinic acid | -6.4938 | Phenolic compounds | (14) Gly173, Asn172, Arg368, Lys382, Val373, Val370, Glu371, Asn223, Pro221, Lys70, Gln224, Gly72, Leu222, Gln73, | (14) |
|  | Podocarpusflavone A | -6.4916 | Flavonoid | (16) Asn172, Ile171, Thr375, Val373, Asn223, Asp372, Gln73, Leu222, Lys70, Gln224, Gly72, Glu371, Pro221, Val370, Pro369, Arg368 | (1) |
|  |  Succedaneaflavanone | -6.4854 | flavanoid | (36) GlnA327, GluA371, SerA71, SerA386, GlyA72, GlnA73, TrpA176, ArgA368, AsnA223, GluA400, LysA70, ProA221, LeuA221, PheA221, ThrA375, GluA326, AsnA294, LeuA323, GlyA219, LeuA324, GlyA173, IleA171, ValA373, AlaA218, Gln A224, GlyA217, ValA370, ProA369, GlyA74, GlyA325, ThrA293, LysA283, AsnA172, GlnA134, AspA372, GlyA291 | (15) |
|  |   Rubrofusarin 6-O-beta-D-Glucopyranoside | -6.4718 | Flavonoid | (13) Phe220, Lys70, Leu222, Asn223, Pro221,Glu371, Aso372, Val370, Leu328, Pro369, Arg368, Lys382, | (12) |
|  |  sebestenoids C | -6.4564 | Flavonoid | (17) Asn223, Leu222,Gln73, Gly173, Gly72, Lys70,Lys70, Gln224, Glu371, Val370, Lys382, Pro369, Glu326, Arg368, Val373, Thr375, Asp372 | (8) |
|  |   liensinine | -6.4458 | Alkaloids | (14) Val373, Asp372, Lys382, Pro369, Arg368, Val370, Glu371, Gln73, Lys70, Leu222, Gly173, Gly72, Gln224, Thr375 | (1) |
|  |   2-Feruloylaloesin | -6.4431 | Flavonoid | (15) Val373, Leu328, Lys382, Arg368, Val370, Glu371, Asn223, Leu222, Gln224, Lys70, GLn73, Gly72, Val227,Gly173, Asn172 | (6) |
|  |   Salvianolic acid C | -6.4398 | Phenolic compounds | (13) Lys168, Gln134, Asn172, Ile171, Gly173, Gly72, Lys70, Gln73, Arg368, Pro369, Val370, Gln224, Asn223 | (16) |
|  |   Palmatine | -6.4249 | Alkaloids | (8)Val352, Arg412, Tyr251, Glu351, Tyr245, Trp250, Leu249, Ser248 | (17) |
|  |   Salvianolic acid B | -6.4199 | Phenolic compounds | (17) Glu326, Gly325, Lys382, Thr293, Asn172, Arg368, Pro369, Lys70, Gln224, Gln73,Val370, Gly72, Leu222, Glu371, Asp372, Val373, Leu328 | (16) |
|  |   4,2,6-trihydroxy-dihydrochalcone-4-O-(6-galloyl)-beta-D-glucopyranose | -6.3546 | Flavonoid | (16) Lys168, Phe170, Phe108, Asn172, Gly173, Ile171, Asn223, Gln224, Gln73, Gly72, Leu222, Lys770, Pro221, Pro369, Arg368, Val370 | (1) |
|  |   didymin | -6.3507 | Flavonoid | (17) Arg296, Lys285, Thr390, Tyr259, Gly95, Ser96, Thr133, Tyr132, Pro131, Glu186, Pro190, Val130, Ile187, Asp191, Arg189, Ala188,Asp192 | (1) |
|  |   Berberine | -6.2667 | Alkaloids | (10) Asp192, Arg189, Glu186, Ile187, Pro131, Val130, Tyr259, Tyr132, Gly95, Ser96 | (17) |
| 50. | Biolobetin | -6.3386 | Flavonoid | (21) ValA373, AspA372, GlnA224, LysA382, LysA70, PheA220, IleA171, ProA221, ThrA375, ArgA368, LeuA222, GluA326, GlnA73,LeuA223, GlyA72, GlyA173, ValA370, AsnA172, ProA369, AsnA223, GluA371 | (1) |
| Standard |   MK8931 | -5.0930 |  |  | (18) |

**References**

1. Murata K. Chemical Diversity of β-Secretase Inhibitors From Natural Resources. Nat Prod Commun. 2019 Dec 1;14(12):1934578X1989481.

2. Bhakta HK, Park CH, Yokozawa T, Tanaka T, Jung HA, Choi JS. Potential anti-cholinesterase and β-site amyloid precursor protein cleaving enzyme 1 inhibitory activities of cornuside and gallotannins from Cornus officinalis fruits. Arch Pharm Res. 2017 Jul 1;40(7):836–53.

3. Youn K, Jun M. In vitro BACE1 inhibitory activity of geraniin and corilagin from geranium thunbergii. Planta Med. 2013;79(12):1038–42.

4. Lee J, Jun M. Dual BACE1 and cholinesterase inhibitory effects of phlorotannins from ecklonia cava-an in vitro and in silico study. Mar Drugs. 2019 Feb 1;17(2).

5. Jung HA, Ali MY, Jung HJ, Jeong HO, Chung HY, Choi JS. Inhibitory activities of major anthraquinones and other constituents from Cassia obtusifolia against β-secretase and cholinesterases. J Ethnopharmacol. 2016 Sep 15;191:152–60.

6. Lv L, Yang QY, Zhao Y, Yao CS, Sun Y, Yang EJ, et al. BACE1 (β-secretase) inhibitory chromone glycosides from Aloe vera and Aloe nobilis. Planta Med. 2008 Apr;74(5):540–5.

7. Naushad M, Durairajan SSK, Bera AK, Senapati S, Li M. Natural Compounds with Anti-BACE1 Activity as Promising Therapeutic Drugs for Treating Alzheimer’s Disease. Vol. 85, Planta Medica. Georg Thieme Verlag; 2019. p. 1316–25.

8. Williams P, Sorribas A, Howes MJR. Natural products as a source of Alzheimer’s drug leads. Vol. 28, Natural Product Reports. 2011. p. 48–77.

9. Choi CW, Choi YH, Cha MR, Kim YS, Yon GH, Hong KS, et al. In vitro BACE-1 inhibitory activity of resveratrol oligomers from the seed extract of paeonia lactiflora. Vol. 77, Planta Medica. 2011. p. 374–6.

10. Sheean P, Rout MK, Head RJ, Bennett LE. Modulation of in vitro activity of zymogenic and mature recombinant human β-secretase by dietary plants. FEBS Journal. 2012 Apr;279(7):1291–305.

11. Abdul B, Vidhyavathi R, Magesh J, Vijayakumar M, Mustafa M, Marikar F. Synthesis and Development of BACE 1 Inhibitor for Alzheimer’s Diseases from Medicinal Plants – Review Article. Annu Res Rev Biol. 2018 Aug 18;28(3):1–18.

12. Youn K, Jun M. Biological evaluation and docking analysis of potent BACE1 inhibitors from boesenbergia rotunda. Nutrients. 2019 Mar 1;11(3).

13. Hwang EM, Ryu YB, Kim HY, Kim DG, Hong SG, Lee JH, et al. BACE1 inhibitory effects of lavandulyl flavanones from Sophora flavescens. Bioorg Med Chem. 2008 Jul 15;16(14):6669–74.

14. Eom TK, Ryu BM, Lee JK, Byun HG, Park SJ, Kim SK. β-secretase inhibitory activity of phenolic acid conjugated chitooligosaccharides. J Enzyme Inhib Med Chem. 2013 Feb;28(1):214–7.

15. Shrestha S, Park JH, Lee DY, Cho JG, Seo WD, Kang HC, et al. Cytotoxic and neuroprotective biflavonoids from the fruit of Rhus parviflora. J Korean Soc Appl Biol Chem. 2012 Aug;55(4):557–62.

16. Yu T, Paudel P, Seong SH, Kim JA, Jung HA, Choi JS. Computational insights into β-site amyloid precursor protein enzyme 1 (BACE1) inhibition by tanshinones and salvianolic acids from Salvia miltiorrhiza via molecular docking simulations. Comput Biol Chem. 2018 Jun 1;74:273–85.

17. Anti-Alzheimer and Antioxidant Activities of Coptidis Rhizoma Alkaloids - Google Search [Internet]. [cited 2020 Jan 23]. Available from: https://www.google.com/search?q=Anti-Alzheimer+and+Antioxidant+Activities+of+Coptidis+Rhizoma+Alkaloids&rlz=1C1GCEA_enPK868PK868&oq=Anti-Alzheimer+and+Antioxidant+Activities+of+Coptidis+Rhizoma+Alkaloids&aqs=chrome..69i57j69i61.1006j0j7&sourceid=chrome&ie=UTF-8

18. Vassar R. BACE1 inhibitor drugs in clinical trials for Alzheimer’s disease. Vol. 6, Alzheimer’s Research and Therapy. BioMed Central Ltd.; 2014.
