# Supplementary material for: Cheminformatics-driven discovery of natural isoquinoline alkaloid inhibitors of Beta-secretase I for Alzheimer’s management
Source: PLoS One. 2026 Mar 2;21(3):e0343717. doi: 10.1371/journal.pone.0343717 (PMC12952630; doi:10.1371/journal.pone.0343717)
Supplement: S3 Table — (DOCX) [file pone.0343717.s003.docx]

**Table S3**. Toxicity analysis of selective compounds by ADMETSAR

| **Compound No.** | **Compound name** | **AMES Toxicity** | **Carcinogenicity** | **P-glycoprotein substrate** |
| --- | --- | --- | --- | --- |
| 45 | Palmatine | No  0.7052 | No  0.9492 | No  0.52 |
| 49 | Berberine | No  0.9132 | No  0.9539 | No  0.6002 |
